# From water to land: evolution of tRNA gene repertoires in photosynthetic organisms

**DOI:** 10.1038/s44318-026-00904-y

**Published:** 2026-08-22

**Authors:** Guillaume Hummel, David Pflieger, Alexandre Berr, Laurence Drouard

**Affiliations:** Institut de biologie moléculaire des plantes, CNRS, Université de Strasbourg, Strasbourg, France

**Keywords:** Evolution & Ecology, Plant Biology, Translation & Protein Quality

## Abstract

Photosynthetic eukaryotes have undergone evolutionary shifts from aquatic to terrestrial habitats, accompanied by changes in genome organization and gene regulation. Yet, the evolution of transfer RNA (tRNA) gene repertoires has received limited attention despite their central role in translation. Here, we review how tRNA gene content, structure, and genomic organization diversified across photosynthetic lineages, mainly Archaeplastida, and how changes relate to evolutionary transitions. We show that tRNA gene repertoires are shaped by ecological transitions, genome architecture, and translational demands. We highlight terrestrialization as a shift in tRNA evolution, marked by loss of selenocysteine and its dedicated tRNA, and changes in intron prevalence and structure. Copy number variation correlates with codon usage and amino acid composition, and in angiosperms, nuclear tRNA genes display reinforced cis-regulatory elements consistent with increased translational demands. We show that plant tRNA genes exhibit evenly dispersed arrangements, except in some algae enriched in clustered configurations. Together, these observations support a model in which tRNA gene repertoires are drivers of genome evolution, integrating translational demand, genomic organization, and ecological adaptation across photosynthetic lineages.

## Abstract (web summary)

This review examines how tRNA gene repertoires diversified across photosynthetic lineages, highlighting how terrestrialization, genome architecture and translational demands have shaped their evolution.

## Graphical abstract

**Figure:**
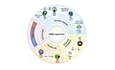

## Introduction

**Figure 1:**
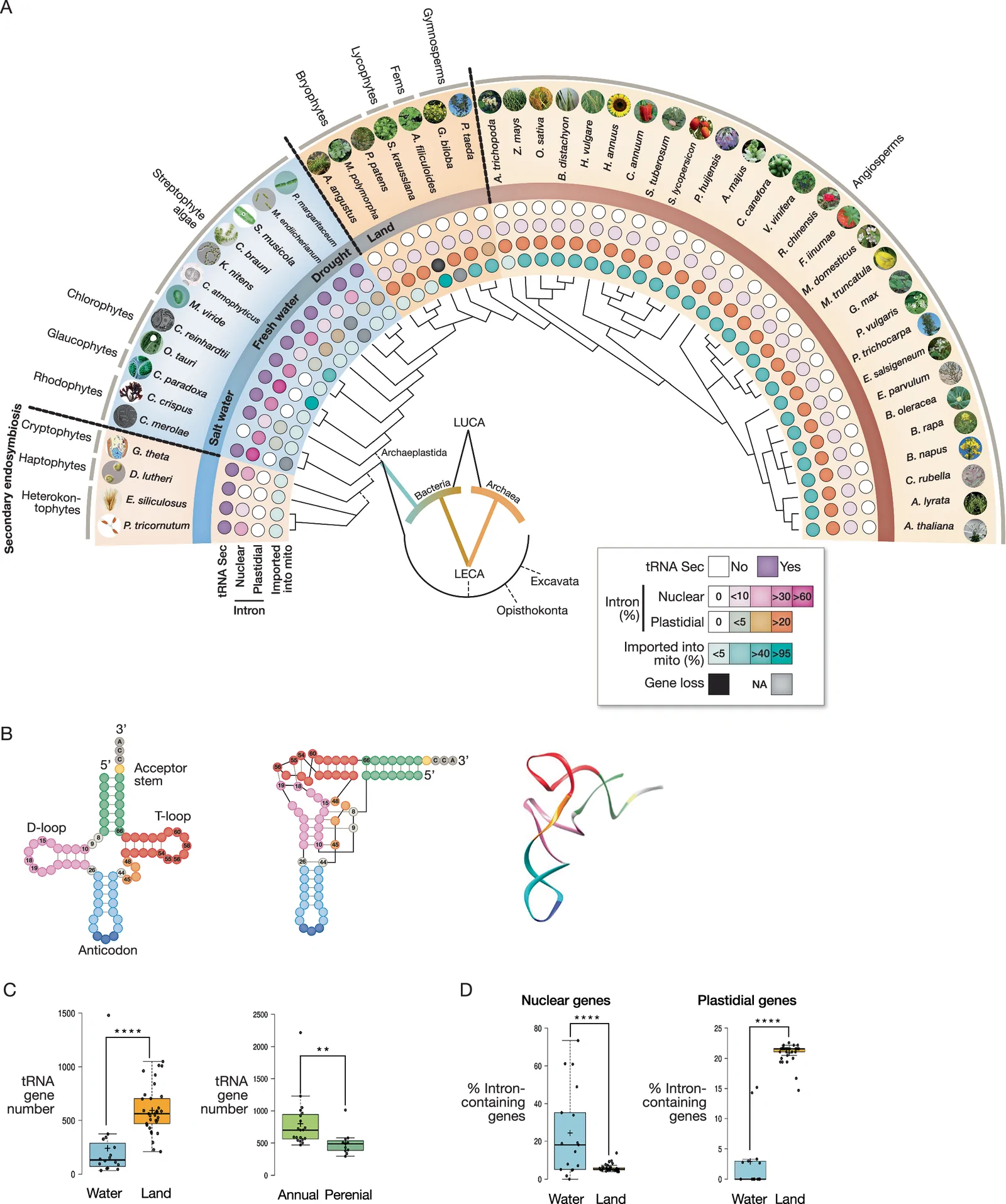
Evolutionary landmarks of tRNA genes in photosynthetic eukaryotes. (**A**) Cladogram adapted from (Cognat et al, 2022), highlighting key evolutionary milestones such as terrestrialization and the emergence of flowering plants. Names of organisms are abbreviated as in Appendix Table S1. The presence of tRNA^Sec^ (Sec), the proportion of tRNAs containing nuclear or plastidial introns, and the percentage of tRNAs predicted to be imported into mitochondria are indicated. Gray circles denote missing data, whereas a black circle indicates a complete loss of the corresponding tRNA class. The emergence of eukaryotes from the last universal common ancestor (LUCA) is depicted as a symbiogenic event between an archaeal host and an alphaproteobacterial endosymbiont (Skejo et al, 2021), giving rise to the Last Eukaryotic Common Ancestor (LECA). Subsequently, the endosymbiosis of a cyanobacterium (green lane) within a eukaryotic host led to the emergence of the Archaeplastida. (**B**) Schematic representation of the 2D (middle panel) and 3D (left panel) structures of a mature tRNA. A ribbon diagram of the 3D structure is shown in the right-bottom panel. (**C**) Box plots displaying total tRNA gene numbers between aquatic (blue) and land (orange) photosynthetic species, and between annual (light green) and perennial (dark green) land plants (including angiosperms and gymnosperms). Data are provided in Appendix Table S1. (**D**) Box plots showing the percentage of intron-containing nuclear or plastid tRNA genes in aquatic (blue) versus land (orange) photosynthetic species. Data are provided in Appendix Table S1. Significance was evaluated using a Mann–Whitney *U* test, (****P ≤ 0.0001, **P ≤ 0.01). All data provided in this figure are from (Cognat et al, 2022).

The colonization of land by plants represents one of the most transformative events in the history of life, profoundly reshaping terrestrial ecosystems (Bar-On et al, 2018). This transition (Fig. 1a) began in aquatic environments ~1.5 billion years ago with the primary endosymbiosis of a cyanobacterium, giving rise to plastids and the green lineage (Chloroplastida). From this ancestor emerged chlorophytes (green algae) and streptophyte algae, among which Zygnematophyceae are now recognized as the closest relatives of land plants (Feng et al, 2024; Bierenbroodspot et al, 2024; Carrillo-Carrasco et al, 2025). Around 500 million years ago, streptophyte algae initiated terrestrialization (Fig. 1a), gradually adapting to desiccation, increased UV radiation, and novel environmental constraints (Domozych and Bagdan, 2022). Early land plants such as bryophytes (hornworts, liverworts, and mosses) retain several ancestral features, including the absence of true vascular tissues and roots (Bowman et al, 2017; Rensing et al, 2008). The appearance of vascular tissues marked a decisive step, with lycophytes and ferns representing the earliest surviving lineages (Banks et al, 2011; Li et al, 2018a). Approximately 350 million years ago, seed plants arose, giving rise to extant gymnosperms (Nystedt et al, 2013). Finally, the emergence of flowers ~150 million years ago drove the ecological dominance of angiosperms, which now comprise ~90% of terrestrial plant species (Zuntini et al, 2024; Amborella Genome, 2013). Despite extensive genomic resources, how these transitions have shaped key molecular systems, including translation, remains only partly understood.

Transfer RNAs (tRNAs) occupy a central position in gene expression by linking codons to amino acids through aminoacylation at their invariant 3’-CCA end and anticodon recognition (Koonin and Novozhilov, 2017; Ribas de Pouplana et al, 2017). Their origin remains debated, but their conserved sequence and structure across all domains of life point to an ancient evolutionary origin, likely predating the last universal common ancestor (Lei and Burton, 2020; Mace and Gillet, 2016; Balke et al, 2015; Tamura, 2015). With rare exceptions in the highly reduced mitochondrial genomes of nematodes or mites (Wende et al, 2013), tRNAs adopt a classical cloverleaf secondary structure and L-shaped tertiary conformation (Fig. 1b), reflecting high evolutionary constraints and a largely monophyletic origin.

During evolution, tRNAs diversified and acquired extensive chemical modifications that stabilize their structure and ensure translational fidelity, particularly within the anticodon loop (Suzuki, 2021; Boccaletto et al, 2022; Ohira and Suzuki, 2024). At the genomic scale, this diversity is reflected by wide variation in tRNA gene copy number, ranging from a dozen in bacteria to several thousand in complex eukaryotes, and by differences among isoacceptors (same amino acid, different anticodons) and isodecoders (same anticodon, different sequences) (Chan and Lowe, 2019; Goodenbour and Pan, 2006; Cognat et al, 2022; Santos and Del-Bem, 2022), raising the possibility of functional specialization (Hughes et al, 2023). Additional variation arises from the presence of introns, which differ widely in number, structure, and position across the tree of life and provide insights into RNA processing and genome evolution (Gozashti et al, 2022; Yoshihisa, 2014). Finally, the genomic organization of tRNA genes represents another key aspect of tRNA biology. Across eukaryotes, tRNA genes can occur as singletons or as clusters (sometimes referred to as “islands”), and may also be embedded within or associated with other genomic elements, including protein-coding genes, retrotransposons, or small non-coding RNA loci (Hani and Feldmann, 1998; Kruszka et al, 2003; Nakaar et al, 1994; Sagi et al, 2016). In angiosperms, tRNA genes are predominantly dispersed, with only limited clustering in specific lineages (Hummel et al, 2020; Monloy and Planta, 2024). Such positional diversity suggests that tRNA gene organization is not random, but instead reflects functional and structural constraints, with potential consequences for RNA polymerase III (Pol III) transcription, local chromatin structure, and nuclear architecture (Ehrlich et al, 2021; Hamdani et al, 2019; Hummel and Liu, 2022; Van Bortle and Corces, 2012).

Despite their ancient origin and central function, the evolutionary dynamics of tRNA gene repertoires have rarely been integrated into the history of plant evolution. Here, we propose that remodeling of tRNA gene complements represents an underappreciated driver of evolutionary innovation across photosynthetic eukaryotes. Lineage-specific reconfigurations, including the loss of selenocysteine and its dedicated tRNA in land plants, contrasting intron patterns across algal lineages, extensive remodeling of mitochondrial and plastid tRNA sets through gene loss and RNA import, and diversification of *cis*-regulatory elements in angiosperms, highlight the remarkable plasticity of tRNA gene systems. In parallel, atypical and permuted tRNA genes observed in certain red algae further point to either retention of ancestral features or convergent evolutionary innovations.

Together, these observations support a model in which tRNA gene repertoires are not passive genomic features, but dynamic components of translational capacity, cellular regulation, and adaptive potential. By integrating comparative genomic and molecular evidence, we aim to reposition tRNA gene evolution as a key contributor to the diversification of photosynthetic life.

## Expansion and diversification of tRNA gene repertoires in photosynthetic lineages

### Absolute variation and extreme cases of tRNA gene numbers

A major dimension of tRNA gene evolution lies in the variation of gene copy number across species. As reported in Hummel et al (2026) and Monloy and Planta (2024), the evolutionary dynamics of tRNA gene repertoires across major photosynthetic lineages, from glaucophytes to angiosperms, including taxa of secondary endosymbiotic origin, reveal wide variation in nuclear tRNA gene number across organisms (Fig. 1A; Appendix Table S1). This number varies from a few dozen in unicellular algae to several thousand in complex plant genomes. This variation broadly reflects differences in genome architecture, organismal complexity, and ecological lifestyle. Aquatic species, typically unicellular or weakly multicellular, generally encode fewer tRNA genes, whereas terrestrial plants display substantially larger repertoires (Fig. 1C; Appendix Table S1). This pattern probably reflects differences in genome architecture, including genome size and duplication history, but may also be consistent with lower translational demands in unicellular or weakly multicellular species. However, these hypotheses should be considered with caution since the dataset underlying these comparisons is enriched in angiosperms, reflecting current genome availability.

Extreme variations further illustrate the dynamic nature of tRNA gene repertoires. For instance, the unicellular streptophyte alga *Penium margaritaceum* exhibits an unusually large number of tRNA genes (~1450) compared to other streptophyte algae (e.g., 96 tRNA genes in *Cholrokybus atmophyticus* and 373 in *Chara braunii*), likely reflecting genome expansion and transposable element (TE) proliferation (Jiao et al, 2020), a mechanism also implicated in the emergence of novel tRNA genes in other eukaryotes, including birds (Ottenburghs et al, 2021). Among land plants, the fern *Azolla filiculoides* represents one of the most extreme cases, harboring more than 7500 tRNA genes, likely reflecting genome duplication and high repeat content (Li et al, 2018a). Its exceptionally rapid growth (Kollah et al, 2016) may benefit from an expanded tRNA pool supporting high translational demand, although a direct causal link remains to be demonstrated. Similarly, the gymnosperm *Pinus taeda* contains ~1000 tRNA genes, consistent with its large, repeat-rich genome and relatively high growth rate among pines (Zimin et al, 2017). In contrast, unicellular algae such as *Ostreococcus tauri* (45 tRNA genes*)* and *Cyanidioschyzon merolae* (43 tRNA genes*)* encode minimal yet sufficient repertoires, consistent with their compact genomes and low TE content (Derelle et al, 2006; Matsuzaki et al, 2004). Together, these examples highlight the potential interplay between genome expansion, transposable element activity, and ecological and physiological constraints in shaping tRNA gene repertoires.

Among organisms with secondary plastids, the haptophyte *Diacronema lutheri* (Hulatt et al, 2021) and the diatom *Phaeodactylum tricornutum* (Bowler et al, 2008) also encode fewer than 60 nuclear tRNA genes. An even more extreme case is the cryptophyte *Guillardia theta*, whose secondary plastid retains a relict endosymbiont nucleus, the nucleomorph (Curtis et al, 2012). Despite its tiny genome (<1 Mb), this nucleomorph encodes 37 tRNA isoacceptors, whereas the nuclear genome encodes 46 (Cognat et al, 2022). Because the nucleomorph is genetically and functionally isolated from the host nucleus (Curtis et al, 2012), it must retain a minimal yet sufficient tRNA set to decode all sense codons (Appendix Fig. S1), likely through flexible wobble pairing (Lei and Burton, 2022; Rogalski et al, 2008).

Variation in tRNA gene number is primarily influenced by genome architecture, including genome size, duplication history, and transposable element content. Nevertheless, physiological and ecological traits may also be associated with some of this variation. For example, within the Lamiales and despite similar genome sizes, the cave plant *Primulina huaijiensis*, which grows slowly in nutrient-poor habitats, encodes half as many tRNA genes as *Antirrhinum majus*, a more typical fast-growing species (Feng et al, 2020; Li et al, 2019). Likewise, fast-growing crops such as sunflower and maize (Andrade et al, 2005) possess more than twice as many tRNA genes as more moderate growers such as grapevine or coffee (Ojeda et al, 2002; DaMatta and Ramalho, 2006). However, these examples are based on a limited sampling of angiosperm species and should therefore be considered illustrative rather than indicative of a general relationship between growth rate and tRNA gene number. Whether similar trends hold across broader angiosperm diversity and other land plant lineages remains to be explored. In duckweeds (Lemnaceae), among the smallest and fastest-growing angiosperms, tRNA gene numbers also vary widely across species (from 237 to 623) (Ernst et al, 2025), highlighting substantial variation even within a relatively homogeneous ecological group. This group may therefore represent an attractive model to investigate the potential links between tRNA gene content and growth-related traits. More broadly, the expansion of tRNA gene numbers in fast-growing species likely reflects underlying genomic processes such as duplication and genome expansion. Whether such variation also contributes to increased translational throughput remains uncertain, although both aspects may interact under selective pressure for efficient translation.

### Translational adaptation and selective constraints

To ensure optimal protein synthesis, tRNAs decoding frequently used codons must not be limiting, whereas excessive tRNAs for rare codons may compromise translational fidelity. Across eukaryotes, tRNA gene copy number is recognized as a proxy for tRNA abundance (Novoa and Ribas de Pouplana, 2012; Kirchner and Ignatova, 2015) and correlates with measured tRNA levels in model systems such as yeast and animals (Behrens et al, 2021; Nagai et al, 2021; Duret, 2000). In addition, tRNA gene copy numbers align with codon usage in highly expressed genes in plants and other eukaryotes (Michaud et al, 2011; Hummel et al, 2026). However, this correspondence is not absolute. In many cases, codon-anticodon imbalances are tolerated and may even be advantageous, as rare codons decoded by low-abundance tRNAs can modulate translation dynamics and promote proper protein folding (Plotkin and Kudla, 2011; Zhou et al, 2013). Consistent with this, excess tRNA gene copies can be locally clustered and subject to epigenetic regulation, allowing context-dependent modulation of tRNA gene expression (Hummel et al, 2020; Zhang et al, 2025). For example, specific tRNA gene clusters in *Arabidopsis thaliana* are transcriptionally repressed or reactivated depending on tissue context (Hummel et al, 2020; Hummel et al, 2025).

Together, these observations suggest that translational imbalance is not merely tolerated but may be selectively maintained to fine-tune translation rates, optimize energy use, and regulate protein maturation. The striking conservation of amino acid frequencies among organisms using the standard genetic code (Tsuji et al, 2010) further indicates that tRNA gene repertoires evolve under strong selective constraints, preserving translational efficiency while allowing subtle regulatory imbalances. Together, these findings support a model of tight co-evolution between codon usage, amino acid composition, and tRNA availability. Overall, tRNA gene repertoires reflect the combined influence of genome dynamics and selective pressures for efficient and adaptable translation.

## The decline of selenocysteine and tRNA^Sec^ in land plants

Beyond the 20 canonical amino acids, incorporation of selenocysteine (Sec) requires a specialized machinery and a dedicated tRNA^Sec^ that recodes UGA stop codons. Selenoproteins are widespread across the three domains of life and contribute to redox homeostasis and cellular defense (Chaudiere, 2023; Lu and Holmgren, 2009). However, their distribution is highly uneven. The Sec machinery and selenoproteins have been repeatedly lost across both prokaryotes and eukaryotes, including in several metazoan (e.g., insects) and fungal lineages (Mangiapane et al, 2014; Mariotti et al, 2019), and are entirely absent from land plants (Lobanov et al, 2007) (Fig. 1A, white circles). In contrast, tRNA^Sec^ is retained in most algal groups, including streptophyte algae, glaucophytes, and species with secondary plastids (Fig. 1A, purple circle), although with a patchy distribution in rhodophytes where both retention and loss of the Sec machinery are observed (Liang et al, 2019; Jiang et al, 2020). Early-diverging extremophiles such as *C. merolae* retain tRNA^Sec^ and components of the Sec insertion pathway but encode few, if any, detectable selenoproteins, whereas more derived florideophytes such as *Chondrus crispus* have lost the entire system (Fig. 1A). This contrast supports multiple independent losses of tRNA^Sec^ and associated selenoproteins, with retention largely confined to early-branching extremophilic taxa.

In species that retain it, tRNA^Sec^ is generally single-copy, as observed in most metazoans, with rare exceptions such as zebrafish (Hatfield et al, 2006). Additional deviations are found in some algal lineages. The haptophyte *D. lutheri* encodes nine tRNA^Sec^ genes out of only 56 nuclear tRNA genes, indicating an unusually large investment in Sec incorporation. In the same lineage, *Emiliania huxleyi* exhibits extreme Sec utilization, harboring the largest known eukaryotic selenoproteome (96 selenoproteins) (Mangiapane et al, 2014), likely reflecting adaptation to selenium-rich marine environments. Whether *D. lutheri* possesses a similarly expanded selenoproteome remains unknown, although its elevated tRNA^Sec^ copy number suggests increased demand for Sec incorporation. Consistent with this, *D. lutheri* and other microalgae produce abundant antioxidant and anti-inflammatory compounds, including catalases and peroxidases, some of which are selenoproteins (Avila-Roman et al, 2021; Wen et al, 2024; Chaudiere, 2023).

In photosynthetic organisms, the presence of a tRNA^Sec^ gene is a reliable genomic marker of a functional selenoproteome and is largely restricted to aquatic lineages, including streptophyte algae, the closest relatives of land plants. Both the Sec insertion machinery and its associated tRNA were lost early during terrestrialization. The selective pressures underlying this transition remain unclear, although differences in selenium availability between aquatic and terrestrial environments likely contributed (Lobanov et al, 2007; Cutter, 1982; Jiang et al, 2020). Additional factors, including redox signaling rewiring or viral interactions, remain speculative (Guillin et al, 2019).

## From LUCA to land plants: evolution of tRNA introns

### Occurrence and processing of tRNA introns

Introns are a common structural feature of tRNA genes across all three domains of life, but their positions, structures, and splicing mechanisms vary markedly. In bacteria and eukaryotic organelles, some tRNA genes harbor self-splicing group I or II introns (Haugen et al, 2005; Lehmann and Schmidt, 2003), typically located at the canonical position between nucleotides 37 and 38, immediately downstream of the anticodon. In archaea, introns occur both at this canonical site and at numerous noncanonical positions, often within D- or T-regions (Yoshihisa, 2014). Some archaeal lineages also harbor permuted or split tRNA genes, in which coding segments are rearranged or separated in the genome (Randau et al, 2005; Fujishima et al, 2009; Chan et al, 2011; Randau and Soll, 2008). In eukaryotes, tRNA introns are predominantly found at the canonical position, although rare exceptions occur in certain unicellular algae (Soma et al, 2007; Cognat et al, 2022; Maruyama et al, 2010).

In archaea and eukaryotes, introns are excised by homologous splicing endonucleases (SENs). Archaeal SENs recognize a bulge-helix-bulge (BHB) motif, whereas eukaryotic enzymes rely on structural features of the anticodon stem-loop. In both systems, tRNA ligases subsequently join the processed exons (Yoshihisa, 2014). While the splicing pathway is well characterized, the functional significance of tRNA introns remains less clear. In *S. cerevisiae*, tRNA introns are dispensable but can modulate growth under specific conditions (Hayashi et al, 2019). Intron removal is essential for generating functional tRNAs and may act as a regulatory checkpoint in translation or other cellular processes. In addition, certain anticodon-modifying enzymes preferentially target intron-containing pre-tRNAs, suggesting roles in tRNA maturation and quality control (Hayashi et al, 2019; Sugahara et al, 2008).

### Intron prevalence in photosynthetic lineages

Photosynthetic eukaryotes exhibit distinct patterns in the frequency of intron-containing tRNA genes (Fig. 1A; Appendix Fig. S2) (Cognat et al, 2022). In the glaucophyte *Cyanophora paradoxa*, only three isoacceptor tRNA genes (~5%) contain canonical introns. This proportion increases in green and red algae, reaching 61% in *C. reinhardtii* and 63% in *C. merolae*, spanning 15 and 16 isoacceptor families, respectively. Such high intron prevalence may be physiologically relevant, although this remains to be experimentally validated. In streptophyte algae, intron prevalence varies considerably, from ~34% in *Klebsormidium nitens* to ~5% in the zygnematophyte *P. margaritaceum*, where introns are restricted to tRNA^Mete^ (elongator) and tRNA^Tyr^ genes. This unusually low level closely mirrors that of land plants (Fig. 1A,D), suggesting that intron streamlining may have accompanied early steps of terrestrialization. We hypothesize that *P. margaritaceum*, which inhabits freshwater environments prone to desiccation, may represent a preadaptation toward rapid and robust tRNA maturation under fluctuating conditions, although this remains to be experimentally validated. Consistent with this pattern, the facultatively terrestrial streptophyte alga *C. atmophyticus* also retains introns only in tRNA^Mete^ and tRNA^Tyr^ genes.

Together, these observations suggest that intron reduction predates the emergence of land plants, although this hypothesis remains difficult to test, and the evolutionary forces underlying this pattern are poorly understood.

### Conserved introns and functional implications

Across eukaryotes, the canonical intron of tRNA^Tyr^ is highly conserved and likely represents one of the most ancient intron-bearing tRNA families. In plants and other eukaryotes, this intron is required for pseudouridylation at U_35_ in the anticodon loop (Johnson and Abelson, 1983; Van Tol and Beier, 1988; Szweykowska-Kulinska and Beier, 1992). Intriguingly, the haptophyte *D. lutheri* lacks introns in all tRNA genes, including tRNA^Tyr^, suggesting a lineage-specific simplification of tRNA processing. Introns in tRNA^Mete^ are less widespread but enriched in primary-plastid lineages (red and green algae and land plants), with the notable exception of the glaucophyte *C. paradoxa*. In humans, 2’-O-methylation at C_34_ (Cm_34_) stabilizes tRNA^Mete^, although this modification occurs on an intron-less tRNA (Vitali and Kiss, 2019). Whether similar mechanisms operate in plants remains unknown. The retention of tRNA^Mete^ introns in photosynthetic lineages may therefore reflect an ancestral eukaryotic feature lost in animals, a requirement for intron-dependent modifications, or lineage-specific adaptation to as-yet unidentified factors.

### Intron diversity

Among photosynthetic eukaryotes, intron diversity is particularly pronounced. Land plants and most streptophyte algae retain only canonical 37/38 introns, whereas chlorophytes and rhodophytes exhibit greater architectural variation. In *C. reinhardtii*, introns are canonical and relatively short, while the ultrasmall green alga *O. tauri* contains unusually long introns (~64 nt) and multiple permuted tRNA genes (Maruyama et al, 2010) (Appendix Fig. S3). The red alga *C. merolae* combines permuted tRNAs with introns at noncanonical positions, including D- and T-loops (Soma et al, 2007). Although *C. crispus* lacks permuted genes, it contains both canonical and numerous atypical introns with predicted BHB-like structures resembling those of archaea and *C. merolae* (Cognat et al, 2022) (Appendix Fig. S4).

The canonical 37/38 intron shared across all domains likely represents an ancient feature inherited from the last universal common ancestor (LUCA) (Sugahara et al, 2008). By contrast, more complex architectures, including permuted tRNAs, multiple introns, and/or noncanonical insertion sites, observed in archaea and early-branching algae may reflect either retention of ancestral features or independent innovations maintained by selective pressures. Their persistence suggests potential regulatory or structural advantages, including expanded RNA modification capacity or tighter control of tRNA maturation. Whether these atypical introns originate from deep common ancestry, convergent evolution, or rare horizontal gene transfer (also referred to as lateral gene transfer) remains unresolved (Keeling and Palmer, 2008; Abby et al, 2012). Notably, their shared reliance on homologous SEN enzymes (Yoshihisa, 2014) supports an evolutionary link between archaeal and algal tRNA processing pathways. Their persistence may also reflect constraints related to genome organization or regulatory control, particularly in compact red algal genomes. Among photosynthetic lineages, red algae are particularly informative, as their intron architectures intersect with broader questions of early eukaryotic evolution and plastid origins.

## Algae at the crossroads of eukaryotic evolution and tRNA gene diversity

### Conflicting phylogenomic signals

**Figure 2:**
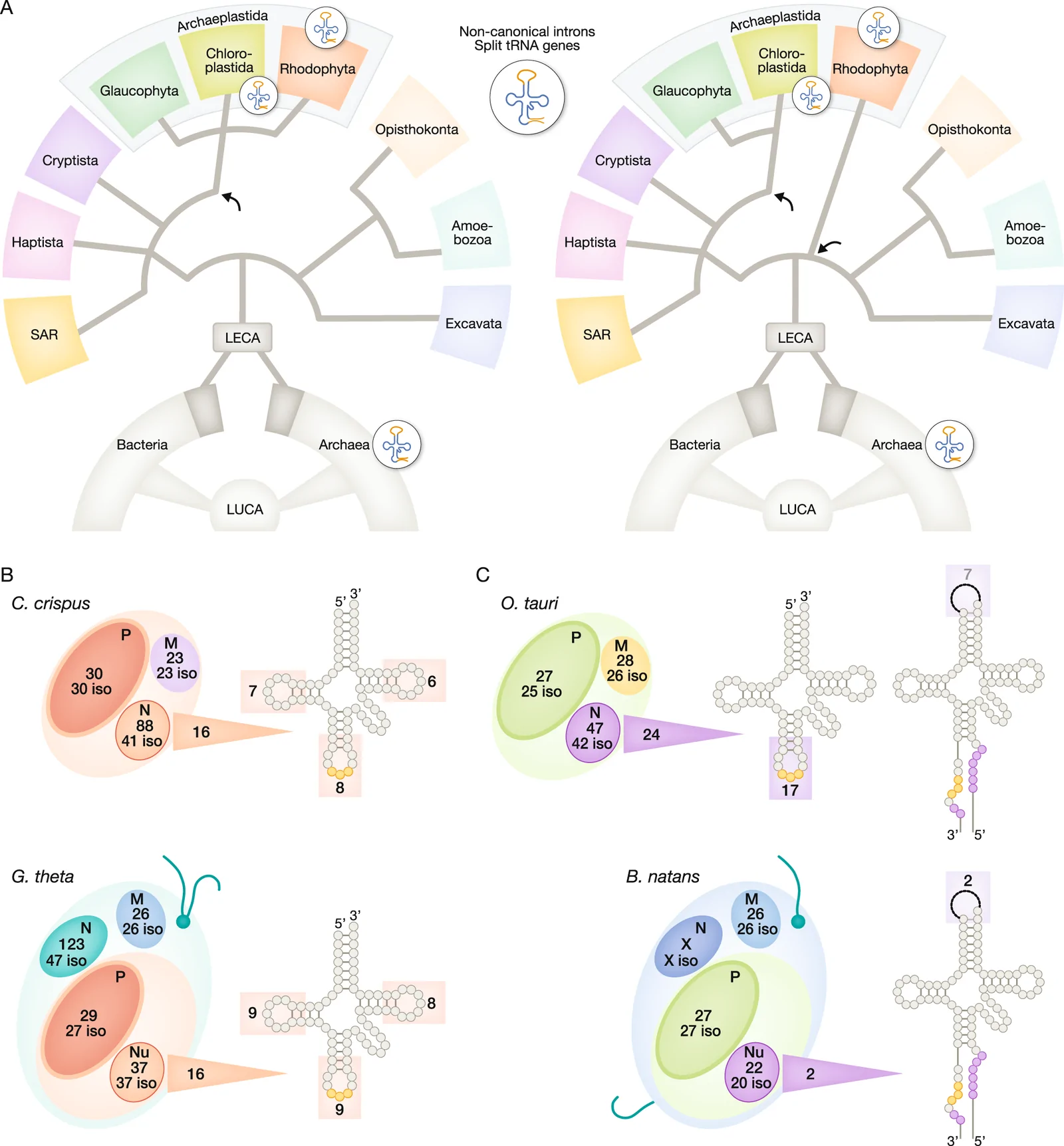
Tracing the origin of red algae and secondary endosymbiotic lineages through tRNA gene evolution. (**A**) Simplified scheme of the eukaryotic tree of life adapted from (Burki et al, 2020). The emergence of eukaryotes is depicted as a symbiogenic event between an archaeal host and an α-proteobacterial endosymbiont (Skejo et al, 2021), giving rise to the Last Eukaryotic Common Ancestor (LECA). Circled tRNAs indicate lineages in which noncanonical introns and split tRNA genes have been identified. While most phylogenomic evidence supports a monophyletic origin of primary plastids in green algae, red algae, and glaucophytes (left), alternative hypotheses propose a polyphyletic origin, with red algae diverging before other Archaeplastida members (right). Green arrows indicate possible endosymbiotic events leading to plastid acquisition. (**B**) Distribution of tRNA genes in the red alga *Chondrus crispus* and the cryptophyte *Guillardia theta*. Numbers indicate total tRNA genes and isodecoders (iso) encoded by the nucleus (N), mitochondrion (M), plastid (P), and nucleomorph (Nu). Red arrowheads denote the number of tRNA genes containing canonical (anticodon loop) and/or noncanonical (D- or T-loop) introns, with their counts shown in the corresponding structural regions (pale red). (**C**) Distribution of tRNA genes in the green alga *Ostreococcus tauri* and the haptophyte *Bigelowiella natans*. The numbers of nuclear, mitochondrial, plastid, and nucleomorph tRNA genes and isodecoders (iso) are shown. Purple arrowheads indicate the number of tRNA genes harboring canonical introns or permuted genes, with their counts under purple background. Intervening sequences are depicted with a black circle. In all structural representations, yellow circles mark the anticodon region, gray circles correspond to nucleotides retained in mature tRNAs, and purple circles indicate nucleotides removed during processing. LUCA Last Universal Common Ancestor, SAR Stramenopiles Alveolates Rhizaria.

Understanding the evolutionary position of red algae is central to reconstructing eukaryotic and plastid evolution (Burki et al, 2020). The origin of primary plastids is generally considered monophyletic, shared by glaucophytes, green plants, and red algae (Fig. 2A), and supported by multiple lines of evidence, including conserved plastid protein import systems, the presence of two cyanobacteria-derived membranes, and congruent phylogenomic signals from plastid and nuclear genes (Rodriguez-Ezpeleta et al, 2005; McFadden and van Dooren, 2004; Archibald, 2009). Nevertheless, some nuclear gene phylogenies have yielded conflicting topologies. For example, actin-based analyses placed *C. crispus* apart, emerging near opisthokonts (Bouget et al, 1995), while phylogenetic analyses on *RBP1* (the largest subunit of RNA polymerase II) suggested that red algae diverged very early, preceding the split of green plants, animals, and fungi (Stiller and Hall, 1997). These discrepancies have prompted alternative scenarios, including multiple primary endosymbiosis events or convergent evolution of plastid features (Palmer, 2003; Stiller and Reel, 2003). Earlier hypotheses proposed that red algae might have arisen from an independent primary endosymbiotic event (Fig. 2A). While such scenario could account for some atypical genomic features, including noncanonical tRNA introns, they remain inconsistent with the strong phylogenomic support for plastid monophyly. This highlights the importance of integrating tRNA gene architecture into broader evolutionary frameworks.

### tRNA architecture and nucleomorph parallels

Atypical tRNA gene architectures in certain red algae provide additional insights into the evolutionary history of tRNA biology. Current models of eukaryogenesis posit that an Asgard archaeal host engulfed an α-proteobacterium, giving rise to the mitochondrion and the last eukaryotic common ancestor (LECA) (Schleper and Sousa, 2020). In this context, similarities between archaeal and algal tRNA processing are more parsimoniously explained by shared ancestry or convergent evolution, rather than direct inheritance from Asgard lineages. Secondary endosymbiotic lineages provide further insights. In the cryptophyte *G. theta*, the nucleomorph genome retains tRNA introns at noncanonical positions (Kawach et al, 2005), resembling those of red algae (*C. crispus*, *C. merolae*) (Soma et al, 2007; Soma et al, 2013), consistent with a red algal endosymbiont origin (Fig. 2B; Appendix Figs. S4 and S5). Conversely, in the chlorarachniophyte *Bigelowiella natans*, permuted tRNA genes similar to those of the green microalga *O. tauri* support a green algal endosymbiont origin (Curtis et al, 2012; Maruyama et al, 2010) (Fig. 2C; Appendix Fig. S3). These examples suggest that nucleomorph genomes have preserved atypical tRNA architectures inherited from their algal ancestors, highlighting their potential as systems to investigate the evolution of tRNA processing and genome organization.

## From endosymbiont to organelle: genomic evolution of tRNA genes in mitochondria and plastids

### Mitochondrial instability and tRNA mosaicism

As descendants of free-living bacteria, mitochondria and plastids now contain highly reduced genomes but retain translation systems that rely on a combination of organelle-encoded and imported components. Because a complete tRNA set is required for translation, the composition and origin of organellar tRNAs provide key insights into genome remodeling over evolutionary time. In most glaucophytes, green and red algae, and streptophyte algae, mitochondrial genomes retain between 20 and 25 tRNA genes inherited from the α-proteobacterial ancestor (Appendix Fig. S6) (Cognat et al, 2022). However, this complement has undergone extensive and lineage-specific reductions. For example, the glaucophyte *C. paradoxa* lacks a mitochondrial tRNA^Thr^ gene, whereas the green alga *C. reinhardtii* encodes only three mitochondrial tRNA genes, and the streptophyte alga *C. atmophyticus* retains 13. Strikingly, although most secondary plastid-bearing lineages, including heterokontophytes, haptophytes, and cryptophytes, have retained the majority of their mitochondrial tRNA genes (Fig. 1A; Appendix Fig. S6a), the mitochondrial genome of *Euglena gracilis*, an excavate flagellate harboring a green plastid acquired through the secondary endosymbiosis of a chlorophyte alga, contains no tRNA genes (Dobakova et al, 2015), suggesting that complete mitochondrial tRNA gene loss has arisen independently in multiple lineages. While the pace of gene loss seems independent of phylogenetic position, it appears to have accelerated with the emergence of land plants, particularly in tracheophytes (Fig. 1A). In angiosperms, mitochondrial genomes contain only about a dozen native tRNA genes, although variation remains substantial. Some lineages, such as *Selaginella*, have lost all of them (Hecht et al, 2011), while others, including *Silene*, retain between 2 and 9 (Sloan et al, 2012; Warren et al, 2021). This variability reflects a rapid and ongoing evolutionary turnover of mitochondrial tRNA gene repertoires. Several non-exclusive factors may account for the faster rate of tRNA gene loss in mitochondria compared to plastids. Plastid gene expression operates under tighter transcriptional and post-transcriptional control due to the high translational demands of photosynthesis, which may impose stronger constraints against gene loss (Zoschke and Bock, 2018). In addition, the extensive structural rearrangements characteristic of plant mitochondrial genomes (Sloan et al, 2012) likely accelerate gene loss through recombination-driven deletions. Together, these factors likely create an asymmetry in the selective pressures acting on mitochondrial versus plastid tRNA gene complements, though their relative contributions remain to be fully disentangled. Regardless of the underlying mechanisms, plant mitochondria compensate for these losses by recruiting tRNAs through three main processes (Appendix Fig. S6). The most widespread is the import of nuclear-encoded tRNAs, a mechanism universal among plants (Duchêne et al, 2009). A second involves intracellular transfers of plastid tRNA genes into mitochondrial genomes. Up to six such genes have been integrated and expressed in angiosperm mitochondria (Maréchal-Drouard et al, 1993). A first instance of plastid-derived tRNA gene insertion, a tRNA^Mete^, was described in the ferns *Ophioglossum californicum* and *Psilotum nudum* (Guo et al, 2017). These transfers appear rare or absent in algal or bryophyte mitochondria (Gandini and Sanchez-Puerta, 2017). A third mechanism, horizontal transfer of bacterial or fungal tRNA genes, has been reported, for example, in the beet *Beta vulgaris* or in the orchid Corallorhiza maculate, respectively (Kubo et al, 2000; Warren et al, 2025). Horizontal transfers appear more common in aquatic and streptophyte algae (Appendix Fig. S6a) (Ma et al, 2022). However, apparent cases of horizontal transfer should be interpreted cautiously, as RNA editing (e.g., C-to-U conversions) can complicate sequence annotation (Lin et al, 1997). Together, these processes illustrate how mitochondrial tRNA repertoires are continuously reshaped by a dynamic balance between gene loss, replacement, and import. An extreme case is *Amborella trichopoda*, whose mitochondrial genome has incorporated numerous foreign sequences, including a large and diverse set of tRNA genes from multiple origins (Amborella Genome, 2013) (Appendix Fig. S7). Despite this abundance, the organelle-encoded tRNA set alone cannot decode all 61 sense codons. For example, it lacks tRNA^Thr^(UGU), required to decode ACA and ACG codons (Alkatib et al, 2012), indicating that mitochondrial translation in this species still depends on tRNA import. A comprehensive understanding of this mosaic tRNA population will require expression-based analyses to distinguish functional genes from pseudogenes. More broadly, functional demonstration of mitochondrial tRNA import requires a combination of organellar RNA fractionation, Northern blot analyses, and, more recently, high-throughput sequencing approaches (Duchêne et al, 2009; Warren et al, 2021). Because a complete set of tRNAs is required to sustain mitochondrial protein synthesis, the import of nuclear-encoded tRNAs is likely essential in plant species that lack many mitochondrially encoded tRNAs. This process also necessitates the import of the full complement of aminoacyl-tRNA synthetases (Duchêne et al, 2005), the enzymes responsible for charging tRNAs with their cognate amino acids prior to their participation in mitochondrial translation. Although not a photosynthetic organism, a striking example of the functional necessity of tRNA import is provided by the protozoan *Trypanosoma brucei*, whose mitochondrial genome completely lacks tRNA genes; inhibition of nuclear-encoded tRNA import results in cell death due to the loss of mitochondrial protein synthesis (Shikha et al, 2020). By contrast, when only a few mitochondrial tRNA genes are absent, their genomic absence alone should not be considered definitive evidence of functional import, and direct experimental validation remains necessary. Although *Amborella trichopoda* represents an exceptional case, its highly chimeric mitochondrial tRNA repertoire highlights the remarkable plasticity of organellar genomes and their capacity to acquire and maintain genetic material from diverse evolutionary origins.

### Relative stability of plastid tRNA repertoires

In contrast to mitochondria, plastid genomes display more stability in their tRNA gene complements. Most plastids encode between 36 and 38 tRNA genes, sufficient to decode all sense codons (Appendix Table S1 and Appendix Fig. S6b), reflecting lower rates of genome rearrangement and gene loss. Nevertheless, several exceptions indicate that plastid tRNA gene repertoires are not entirely static. In some lineages, specific tRNA genes have been lost and functionally compensated for by the import of nuclear-encoded tRNAs. This is particularly evident in lycophytes such as *Selaginella*, which possess reduced and dynamic plastid genomes and rely on imported tRNAs (Berrissou et al, 2024; Shim et al, 2021). Similar losses occur in ferns (Du et al, 2022). For example, *A. filiculoides* lacks tRNA^Lys^(TTT), tRNA^Gly^(TCC), and tRNA^Thr^(TGT) genes (Cognat et al, 2022). Comparable losses are observed in the streptophyte alga *K. nitens*, which lacks plastid-encoded tRNA^Thr^ and tRNA^Val^, and in parasitic or non-photosynthetic angiosperms such as *Epifagus virginiana* (dePamphilis and Palmer, 1990) and the orchid *Rhizanthella gardneri* (Delannoy et al, 2011). Collectively, these cases indicate that plastid tRNA import is more widespread than previously thought and likely originated early in plant evolution. Although rare, horizontal transfer of tRNA genes into plastids has also been documented. In *Mesotaenium endlicherianum*, for example, a plastid tRNA^Lys^(CTT) gene appears to derive from a bacterial tRNA^Asn^(GTT) gene (Rice and Palmer, 2006), consistent with broader bacterial-to-charophyte gene transfers observed in nuclear genomes. Some tRNA genes of unknown origin are also found. For example, it is the case for genes encoding a tRNA^Ala^(GGC) in several streptophyte algae or a tRNA^Pro^(GGG) in gymnosperms or in the streptophyte alga *M. endlicherianum* (Appendix Fig. S6b). Whether these genes are expressed remains to be studied. Unlike mitochondria, plastids have retained intron-containing tRNA genes (Appendix Fig. S2) (Zoschke et al, 2010). In the glaucophyte *C. paradoxa*, a group I intron in tRNA^Leu^(TAA) gene persists across many streptophyte algae and land plants but has been lost in green and red algae and in secondary plastids (Paquin et al, 1997). In the red alga *C. crispus*, a group II intron encoding a maturase interrupts the tRNA^Mete^ gene, a feature restricted to florideophytes (Janouskovec et al, 2013). More broadly, the acquisition and retention of plastid tRNA introns appear to have intensified during the transition to land (Fig. 1A). For example, *P. margaritaceum* shares five intron-containing plastid tRNA genes with modern angiosperms (Appendix Fig. S2), supporting the close evolutionary relationship between Zygnematophyceae and land plants (Jiao et al, 2020). Interestingly, the increase in plastid tRNA introns coincides with a decrease in nuclear tRNA introns (Fig. 1A,D), suggesting a reconfiguration of intron distribution during terrestrialization (Manhart and Palmer, 1990). Together, these observations highlight contrasting evolutionary trajectories: mitochondrial tRNA gene repertoires remain highly dynamic, shaped by continuous loss, replacement, and import, whereas plastid tRNA gene complements are comparatively stable, with deviations primarily associated with parasitism, genome reduction, or terrestrial adaptation.

## Evolution of *cis*-regulatory elements in nuclear tRNA gene transcription

**Figure 3:**
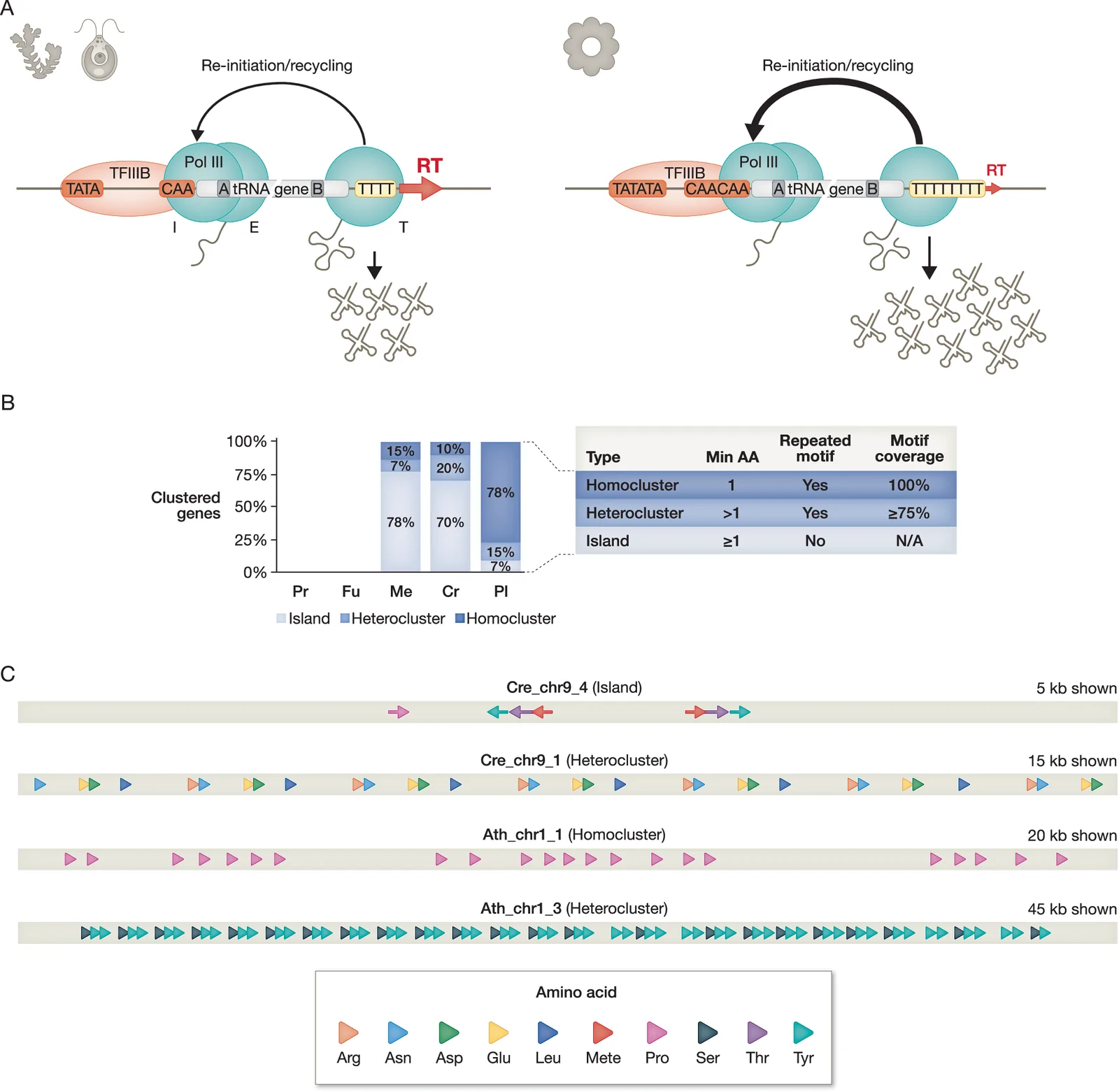
*Cis*-regulatory elements and clustering features of nuclear tRNA genes. (**A**) Conceptual model illustrating how *cis*-regulatory elements may modulate Pol III transcription and recycling efficiency. Core promoter elements include the internal A- and B-boxes, upstream AT-rich and CAA motifs, and downstream poly(T) termination signals. In angiosperms (right), enrichment of CAA motifs and extended poly(T) tracts is associated with enhanced transcriptional efficiency and re-initiation, whereas more basal or motile organisms (left) display simpler architectures supporting constitutive expression. High T content may also limit transcriptional readthrough (RT). I initiation, E elongation, T termination. (**B**) tRNA gene clusters across eukaryotes. Clusters are here defined as groups of ≥5 genes located within a window of 5 kb. Three main configurations are distinguished: homoclusters (single isotype family), heteroclusters (at least 2 isotype families), and islands (large mixed clusters involving multiple tRNA families with diverse transcriptional orientations). Clustering is generally limited in plants, whereas metazoans and some algae display more extensive multi-family clustering. Pr Protists (*C. merolae, P. tricornutum, D. discoidum, and O. tauri*), Fu Fungi (*S. cerevisiae*), Me Metazoans (*C. elegans, D. melanogaster, M. musculus*, and *H. sapiens*), Cre *C. reinhardtii*, Pl land plants (Appendix Table S1). Data are derived from Hummel et al (2026). (**C**) Representative examples of tRNA gene clustering organization. An island configuration is shown in *C. reinhardtii* (Cre). Heteroclusters are illustrated in both *C. reinhardtii* and *A. thaliana* (Ath), while a homocluster is depicted in *A. thaliana*. Arrows indicate transcriptional orientation. Amino acids are color-coded. Figure 3 c was generated using ShinytRNA (https://nebula.ibmp.unistra.fr/shinytRNA/; Hummel et al, 2026).

In eukaryotes, nuclear tRNA genes are transcribed by Pol III (Fig. 3a). Transcription depends on two universally conserved internal promoter elements, the A- and B-boxes, located within the D- and T-stem loops of the tRNA. These motifs are recognized by TFIIIC, which recruits TFIIIB to a TATA-like or AT-rich region, typically within 50 nt upstream of the transcription start site (Michaud et al, 2011). TFIIIB then enables Pol III recruitment and the initiation of transcription. Additional *cis*-regulatory elements further modulate transcription efficiency. Conserved CAA triplets positioned between positions −10 to −3 enhance Pol III transcription in land plants and yeast (Choisne et al, 1998; Yukawa et al, 2000; Giuliodori et al, 2003; Yukawa et al, 2011; Michaud et al, 2011; Monloy and Planta, 2024). Short poly(T) tracts on the non-template strand serve as termination signals (Dieci et al, 2007; Girbig et al, 2022; Lin-Marq and Clarkson, 1998; Platt, 1986; Xie et al, 2022). Although A- and B-boxes are universal, flanking regulatory sequences show clear lineage-specific variation. As detailed in Hummel et al (2026), analysis of upstream (50 nt) and downstream (25 nt) regions across photosynthetic lineages revealed a strongly conserved AT-rich region (−20 to −35) in land plants, particularly angiosperms. In contrast, algae and non-flowering lineages display weaker AT enrichment, resembling non-plant eukaryotes (Zhang et al, 2011; Hummel et al, 2026). Similarly, CAA triplets are prominent in flowering plants and detectable, albeit at a lower frequency, in the earliest-diverging angiosperms *A. trichopoda* (Amborella Genome, 2013). Downstream regions also diverge, with angiosperms containing longer poly(T) tracts (~14 T residues within 25 nt) compared to ~8 in algae and non-flowering plants. Together, as reported in Hummel et al (2026), these patterns suggest progressive reinforcement of Pol III regulatory motifs during angiosperm evolution.

These *cis*-elements (AT-rich regions, CAA triplets, and extended poly(T) tracts) enhance Pol III transcription and efficient re-initiation (Ulmasov and Folk, 1995; Yukawa et al, 2000; Yukawa et al, 2013; Dieci and Sentenac, 1996; Mishra et al, 2021). Their enrichment in angiosperms may reflect adaptation to increased translational demands, particularly during phases of rapid growth such as flowering (Alvarez-Urdiola et al, 2025). Enhanced upstream motifs may strengthen transcription initiation, while extended poly(T) tracts ensure efficient termination and polymerase re-initiation. Maintaining this balance is crucial, as insufficient termination can lead to Pol III readthrough, generating aberrant transcripts or interfering with neighboring genes (Turowski et al, 2016) (Fig. 3A).

## Chromosomal organization and clustering of tRNA genes in the green lineage

### Global organization: insights from Arabidopsis and beyond

The increasing availability of high-quality tRNA gene annotations enables a comparative view of their chromosomal organization across plant genomes. In *A. thaliana*, excluding a few dense clusters (Hummel et al, 2020), tRNA genes are distributed across chromosomes with relatively uniform intergenic distances (Hummel et al, 2026). This pattern is conserved in angiosperms (Monloy and Planta, 2024) and more broadly across land plants (Hummel et al, 2026), indicating a predominantly dispersed organization that is largely independent of total tRNA gene copy number. Clusters therefore represent localized exceptions (Fig. 3B), generating regional increases in tRNA gene density without disrupting overall chromosomal dispersion. By contrast, the green alga *C. reinhardtii* exhibits a more heterogeneous organization (Hummel et al, 2026), resembling patterns described in metazoan genomes (Merchant et al, 2007).

Across photosynthetic eukaryotes, total tRNA gene number shows little or no correlation with genome size (Mohanta et al, 2020; Hummel et al, 2026). In contrast, genome size correlates positively with the median distance between consecutive tRNA genes (Hummel et al, 2026), suggesting that genome expansion is primarily accommodated through increased intergenic spacing rather than proportional amplification of tRNA gene copy number. As further shown in (Hummel et al, 2026; Zhang et al, 2025), analyses of isoacceptor distributions, excluding clustered loci, reveal chromosome-specific enrichments or depletions, indicating that tRNA gene organization is not entirely random at the chromosomal scale. These patterns are consistent with local duplication and turnover processes, although the relative contribution of different mechanisms remains difficult to disentangle. Despite these local variations, overall tRNA gene distribution remains globally balanced, suggesting long-term compensation of duplication events that prevents large-scale positional biases.

Finally, although tRNA genes are broadly distributed along chromosome arms, they are consistently absent from centromeric regions (Hummel et al, 2026). This exclusion is conserved across plant lineages with diverse centromere architectures: compact in *A. thaliana* (Round et al, 1997), repeat-rich in cereals (*Brachypodium distachyon* and *Zea mays*) (Li et al, 2018b; Wolfgruber et al, 2009), and mixed (repeat-based and repeatless) or pericentromeric heterochromatic in Solanaceae (*Solanum tuberosum* and *S. lycopersicum*) (Gong et al, 2012; Demirci et al, 2017). These observations suggest that in plants, stable Pol III transcription of dispersed tRNA genes is likely incompatible with heterochromatic environments. However, 5S rRNA genes constitute a striking exception. In *A. thaliana*, they are organized as long tandem arrays located in pericentromeric heterochromatin, and only a small subset of these repeats forms local decondensed chromatin domains that permit Pol III transcription (Mathieu et al, 2003; Douet and Tourmente, 2007). In yeast, Pol III also displays particular chromatin-associated properties, as tRNA genes can localize at or near heterochromatin boundaries and Pol III transcription complexes can act as barriers that limit the spread of silencing (Noma et al, 2006; Scott et al, 2007; Simms et al, 2008; Van Bortle and Corces, 2012). Such function has not been described in plants.

### Family- and lineage-specific clustering patterns

tRNA genes are predominantly organized as dispersed singletons across eukaryotes. In protists and fungi, this organization is nearly exclusive (Fig. 3B). Alongside this dominant pattern, tRNA genes can also occur in grouped arrangements. These grouped configurations include two main organizational modes (Fig. 3C). The first corresponds to repeated homo- or heteroclusters, in which one tRNA family (homoclusters) or a limited number of isotype families (heteroclusters) are repeated, often in tandem, such as Pro homoclusters or Ser-Tyr-Tyr heteroclusters in *A. thaliana* (Hummel et al, 2020). The second corresponds to larger multi-family assemblies, referred to as tRNA gene islands, where several tRNA gene isotype families are located in close proximity without obvious repeated motifs and often with variable transcriptional orientations. In metazoans, grouped tRNA genes represent only a minor fraction of the total repertoire, but when present they are predominantly organized as large multi-family islands, whereas compact homo- and heteroclusters remain less frequent (Fig. 3B) (Iben and Maraia, 2014; Coughlin et al, 2009). A similar pattern is observed in the green alga *C. reinhardtii* (Fig. 3B,C) (Cognat et al, 2008; Merchant et al, 2007). This organization reflects retention of an ancestral state or convergent evolution toward similar chromatin or transcriptional constraints remains unclear. Land plants show only a small fraction of grouped tRNA genes, and these are mainly represented by repeated homo- and heteroclusters (Fig. 3B,C) (Monloy and Planta, 2024; Zhang et al, 2025; Hummel et al, 2026). In dicots, recurrent clusters involve proline in homoclusters and serine and tyrosine in heteroclusters (Fig. 3C). Other isotypes, such as tRNA^Gln^ genes, may also cluster in specific taxa, while lineage-specific patterns, including tRNA^Ala^ or tRNA^Ile^ gene clusters in some monocots and Brassicaceae, further highlight the diversity of tRNA gene organization (Monloy and Planta, 2024; Zhang et al, 2025; Hummel et al, 2026).

Together, these observations define a continuum of tRNA gene organization across eukaryotes, from predominantly dispersed singletons to large multi-family islands. Land plants occupy an intermediate position within this continuum, with globally dispersed tRNA genes and limited grouping restricted to specific isotype-enriched clusters.

## Concluding remarks, limitations, and future perspectives

**Figure 4:**
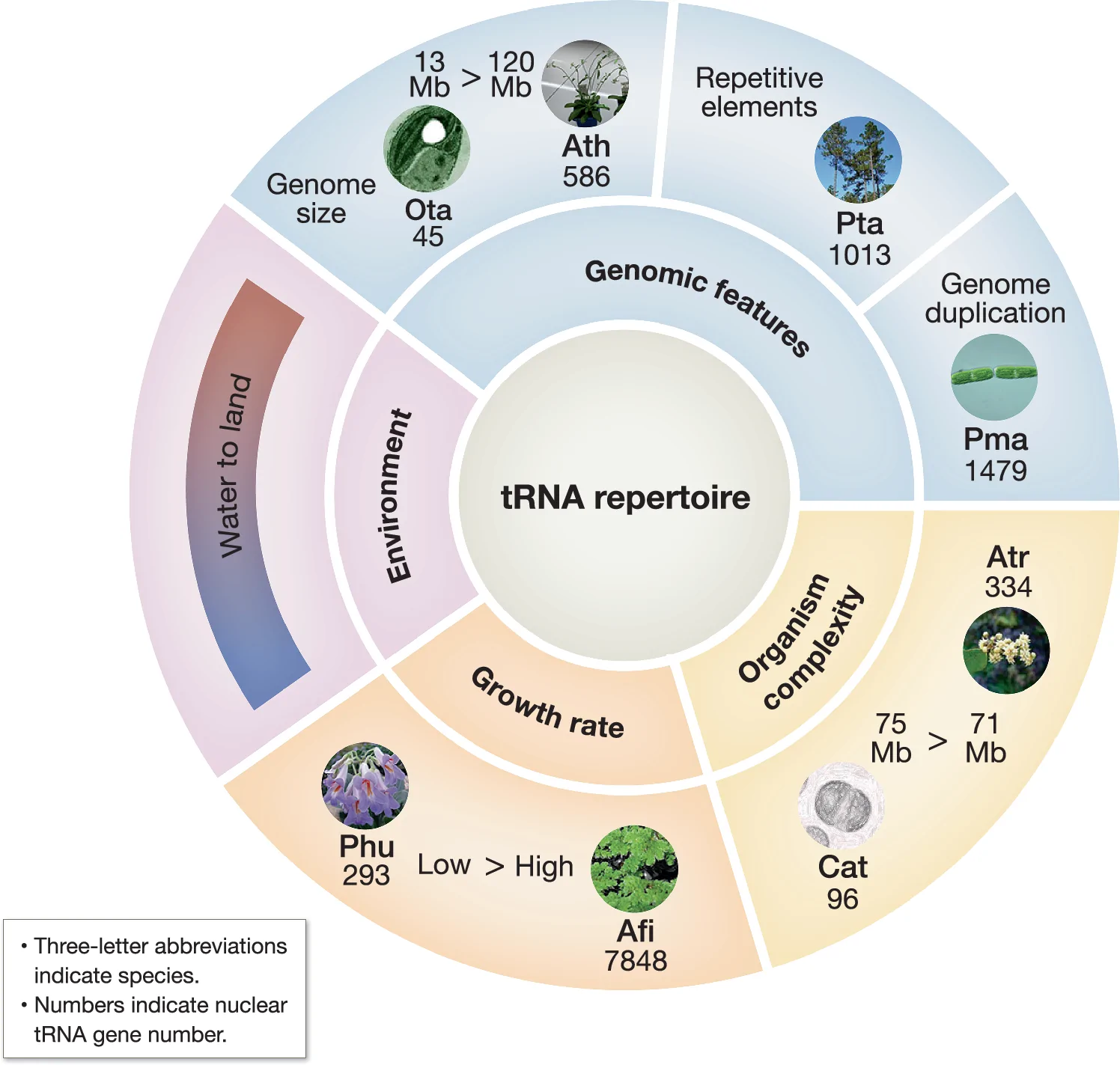
Evolutionary drivers of tRNA gene repertoires in photosynthetic organisms. Schematic overview of the main evolutionary drivers shaping tRNA gene populations, with illustrative examples. Where relevant, the number of nuclear tRNA genes per species is provided. Names of organisms are abbreviated as in Appendix Table S1.

The evolution of tRNA gene repertoires across photosynthetic eukaryotes reveals a complex interplay between genome dynamics and translational constraints (Fig. 4). A central and conserved principle emerges: tRNA gene copy number covaries with codon usage, reflecting a universal constraint linking genome composition to translational demand. However, this relationship is likely shaped by multiple layers of genomic organization, including genome size, duplication history, and transposable element activity, which together drive substantial variation across lineages.

Major evolutionary transitions further illustrate the plasticity of tRNA gene systems. The loss of tRNA^Sec^ gene in land plants, the diversification of intron architectures, and the extensive remodeling of organellar tRNA gene repertoires highlight the dynamic nature of tRNA gene evolution across photosynthetic lineages. Despite this diversity, these changes remain embedded within the conserved functional requirements of the translational machinery.

At the regulatory level, the reinforcement of Pol III *cis*-regulatory elements in angiosperms suggests an additional layer of control that may contribute to fine-tuning transcriptional output during complex developmental transitions. This regulatory evolution complements structural changes in tRNA gene repertoires and points to coordinated adaptation of both gene content and transcriptional efficiency.

At the chromosomal scale, distinct organizational strategies further illustrate lineage-specific solutions to these constraints. Land plants predominantly exhibit dispersed tRNA gene distributions with limited clustering, whereas metazoans and Chlamydomonas more often display aggregated tRNA genes in large mixed-family islands. These contrasting architectures likely reflect different balances between transcriptional coordination and regulatory control, with dispersed organization minimizing local transcriptional interference and maintaining balanced isoacceptor expression, whereas clustered arrangements may promote coordinated Pol III activity within defined chromatin domains.

Future work should clarify the functional significance of these patterns. Whether tRNA gene clusters act as adaptive transcriptional hubs, structural by-products of chromatin organization, or responses to developmental and environmental cues remains unresolved. Integrating chromatin organization, Pol III occupancy, and tRNA gene expression data will be essential to link genome structure with translational output. Recent advances in high-throughput tRNA sequencing technologies (Padhiar et al, 2024), (Marlow and Su, 2026; Alarcon et al, 2026) now provide powerful opportunities to connect genomic tRNA repertoires with expressed tRNA pools. These approaches will be critical for characterizing functional tRNA landscapes across taxa and for deciphering how isodecoder diversity, RNA modifications, and tRNA processing collectively shape translational regulation. The functional roles of tRNA introns, still poorly understood in many photosynthetic lineages, represent another important frontier, as intron presence may influence both modification and folding dynamics.

Together, these findings position tRNA gene evolution as both a marker and a potential contributor to eukaryotic innovation, shaped by universal translational constraints yet implemented through lineage-specific genomic and regulatory architectures.

At the same time, several limitations should be considered when interpreting the patterns discussed in this review. First, the dataset underlying quantitative comparisons (Appendix Table S1) is enriched in angiosperms, reflecting the current availability of high-quality annotated plant genomes. Consequently, inferences regarding non-angiosperm land plants, gymnosperms, and non-green algae remain tentative, and broader taxonomic sampling will be required to assess the generality of the observed patterns. Second, tRNA gene annotations rely primarily on the PlantRNA 2.0 database (Cognat et al, 2022). Although this resource represents the current reference framework for plant tRNA annotation, predictions remain sensitive to annotation parameters and may include pseudogenes, while experimental validation is still limited for most non-model species. Third, tRNA gene copy number is used throughout this review as a proxy for tRNA abundance, despite the fact that this relationship has been rigorously demonstrated in only a limited number of model organisms. Characterizing the expressed functional tRNA pools across diverse plant lineages, as discussed above, represents a critical next step. Finally, our synthesis focuses on the evolution of tRNA gene repertoires at the genomic level and does not systematically address tRNA modifications, which constitute a major and rapidly expanding dimension of tRNA biology. Readers interested in this aspect are referred to recent dedicated reviews (Suzuki, 2021; Ohira and Suzuki, 2024). Our knowledge of tRNA modifications in photosynthetic organisms remains extremely limited with only scattered reports available for a handful of species (Sun et al, 2019; Jin et al, 2019), (Dannfald et al, 2021; Sharma et al, 2023). Advancing this field will be a key research priority in the coming years, driven by the synergistic use of classical analytical methods, such as mass spectrometry, together with rapidly evolving high-throughput technologies. Addressing these limitations through expanded phylogenetic sampling, improved functional validation, and integration of epitranscriptomic data will be essential for developing a more comprehensive understanding of tRNA evolution and function across photosynthetic eukaryotes.

## Supplementary information

Appendix

Peer Review File

## Data Availability

This study includes no data deposited in external repositories.
