# Supplementary material for: From water to land: evolution of tRNA gene repertoires in photosynthetic organisms
Source: EMBO J. 2026 Aug 22;45(18):6367–83. doi: 10.1038/s44318-026-00904-y (PMC13578239; doi:10.1038/s44318-026-00904-y)
Supplement: Supplementary file 1 [file 44318_2026_904_MOESM1_ESM.pdf]

Appendix for

From water to land: evolution of tRNA gene repertoires in photosynthetic organisms

Table of contents:

Appendix Figure S1 (page 2). Genetic code and probable codon/anticodon recognition by *Guillardia theta.*

Appendix Figure S2 (page 3). Nuclear-encoded tRNA genes with introns.

Appendix Figure S3 (page 4). Secondary structures of *Ostreococcus tauri* pre-tRNAs.

Appendix Figure S4 (page 5). Secondary structures of *Chondrus crispus* pre-tRNAs.

Appendix Figure S5 (page 6). Secondary structures of *G. tetha* pre-tRNAs.

Appendix Figure S6 (page 7). Organellar tRNA genes.

Appendix Figure S7 (page 8). Mitochondrial tRNA genes of *Amborella trichopoda*.

Appendix Table S1 (page 9). Photosynthetic species analyzed in this study.

Appendix Figure S1. Genetic code and probable codon/anticodon recognition by *Guillardia theta* (**A**) nucleus- and (**B**) nucleomorph- tRNA genes. iM (initiator Methionine), M (elongator Methionine).

a

aa	codon	tRNA	aa	codon	tRNA	aa	codon	tRNA	aa	codon	tRNA
F	UUU	GAA	L	CUU	AAG	I	AUU	GAU	V	GUU	GAC
F	UUC	GAA	L	CUC	AAG	I	AUC	GAU	V	GUC	GAC
L	UUA	UAA	L	CUA	UAG	I	AUA	UAU	V	GUA	UAC
L	UUG	CAA	L	CUG	CAG	Mi	AUG	CAU	V	GUG	CAC
						Me	AUG	CAU			
S	UCU	AGA	P	CCU	AGG	T	ACU	AGU	A	GCU	AGC
S	UCC	AGA	P	CCC	UGG	T	ACC	AGU	A	GCC	AGC
S	UCA	UGA	P	CCA	UGG	T	ACA	UGU	A	GCA	UGC
S	UCG	CGA	P	CCG	CGG	T	ACG	CGU	A	GCG	CGC
Y	UAU	GUA	H	CAU	GUG	N	AAU	GUU	D	GAU	GUC
Y	UAC	GUA	H	CAC	GUG	N	AAC	GUU	D	GAC	GUC
*	UAA		Q	CAA	UUG	K	AAA	UUU	E	GAA	UUC
*	UAG		Q	CAG	CUG	K	AAG	CUU	E	GAG	CUC
C	UGU	GCA	R	CGT	ACG	S	AGT	GCU	G	GGU	GCC
C	UGC	GCA	R	CGC	ACG	S	AGC	GCU	G	GGC	GCC
*	UGA		R	CGA	UCG	R	AGA	UCU	G	GGA	UCC
W	UGG	CCA	R	CGG	CCG	R	AGG	CCU	G	GGG	CCC

b

aa	codon	tRNA	aa	codon	tRNA	aa	codon	tRNA	aa	codon	tRNA
F	UUU	GAA	L	CUU	AAG	I	AUU	AAU	V	GUU	AAC
F	UUC	GAA	L	CUC	AAG	I	AUC	AAU	V	GUC	AAC
L	UUA	UAA	L	CUA	UAG	I	AUA	UAU	V	GUA	UAC
L	UUG	CAA	L	CUG	UAG	Mi	AUG	CAU	V	GUG	UAC
						Me	AUG	CAU			
S	UCU	AGA	P	CCU	AGG	T	ACU	AGU	A	GCU	UGC
S	UCC	AGA	P	CCC	AGG	T	ACC	AGU	A	GCC	UGC
S	UCA	AGA	P	CCA	AGG	T	ACA	UGU	A	GCA	UGC
S	UCG	CGA	P	CCG	CGG	T	ACG	CGU	A	GCG	UGC
Y	UAU	GUA	H	CAU	GUG	N	AAU	GUU	D	GAU	GUC
Y	UAC	GUA	H	CAC	GUG	N	AAC	GUU	D	GAC	GUC
*	UAA		Q	CAA	UUG	K	AAA	UUU	E	GAA	UUC
*	UAG		Q	CAG	CUG	K	AAG	CUU	E	GAG	UUC
C	UGU	GCA	R	CGT	ACG	S	AGT	GCU	G	GGU	GCC
C	UGC	GCA	R	CGC	ACG	S	AGC	GCU	G	GGC	GCC
*	UGA		R	CGA	UCG	R	AGA	UCU	G	GGA	UCC
W	UGG	CCA	R	CGG	UCG	R	AGG	CCU	G	GGG	UCC

Appendix Figure S2. tRNA genes with introns. **a**) Nuclear-encoded tRNA genes with introns. White: no intron, orange: all tRNAs charging the same amino acids have introns, pale orange: not all tRNAs charging the same amino acid have introns. The numbers of introns out of the total number of tRNA genes for the corresponding amino acid are indicated. **b**) Plastidial-encoded tRNA genes with introns. White: no intron, green: with intron, black: no tRNA gene.
Amino acids charging tRNAs (isotypes) are with the one-letter amino acid code, and elongator (Me) and initiator (Mi) methionine have been differentiated. *The plastidial tRNA gene population of *Chara vulgaris* (Cvu; accession number DQ229107) has been analyzed instead of *C. brauni* (not available).
Names of organisms are abbreviated as in Table S1.

a

	tRNA isotypes																					
Species	A	C	D	E	F	G	H	I	K	L	Me	Mi	N	P	Q	R	S	T	V	W	Y	Number of AA
Ath																						2
Aly																						2
Cru																						2
Bna																						2
Bra																						2
Bol																						2
Epa																						2
Esa																						2
Ptr																						2
Pvu																						2
Gma																						2
Mtr																						2
Mdo																						2
Fii																						2
Rch																						2
Vvi																						2
Cca																						2
Ama																						2
Phu																						2
Sly																						2
Stu																						2
Can																						2
Han																						2
Hvu																						2
Bdi																						2
Osa																						2
Zma																						2
Atr																						2
Pta																						2
Gbi								6/22					1/19									4
Afi																						2
Smo																						2
Ppa																						2
Mpo																						2
Aan																						2
Pma																						2
Men						8/16		1/7	2/9							2/13	3/11	2/8				9
Smu								3/9														4
Cbr								5/17	4/17							2/39		4/18				6
Kni				1/4		5/7		1/3		5/7				2/3		2/7		3/6				12
Cat																						2
Mvi				1/22		8/10			1/5	1/23				3/6		8/9						15
Cre	34/39		15/17	15/20		27/31		1/10	4/15	18/23				12/20		9/22		8/12				15
Ota						1/3				2/4						2/4	2/3	1/3				15
Cme						2/4			1/2	2/4								1/3				16
Ccr	5/9								1/3	1/5				1/5		4/7	3/8	1/6				12
Cpa																	2/6					3
Gth																						1
Dlu																						0
Esi																						1
Ptri				1/3											1/2	2/3	1/4		1/3			9

b

	tRNA Isotypes						
	A	G	I	L	K	V	Me
Ath							
Aly							
Cru							
Bna							
Bra							
Epa							
Ptr							
Pvu							
Gma							
Mtr							
Fii							
Rch							
Vvi							
Cca							
Phu							
Sly							
Stu							
Can							
Han							
Hvu							
Bdi							
Osa							
Zma							
Atr							
Pta							
Gbi							
Afi							
Skr							
Ppa							
Mpo							
Aan							
Pma							
Men							
Cvu*							
Kni							
Cat							
Mvi							
Cre							
Ota							
Cme							
Ccr							
Cpa							
Gth							
Dlu							
Esi							
Ptri							

Appendix Figure S3. Secondary structures of *Ostreococcus tauri* pre-tRNAs. Predicted secondary structures of pre-tRNA^{Arg} and pre-tRNA^{Asn} with permuted introns of archaeal-type are schematically shown. The anticodon is depicted with orange circles. Blue circles represent intervening sequence and purple circles flanking sequences. BHB motifs of archaeal-type introns are under orange background. Arrowheads indicate cleavage sites. BHB: Bulge-Helix-Bulge.

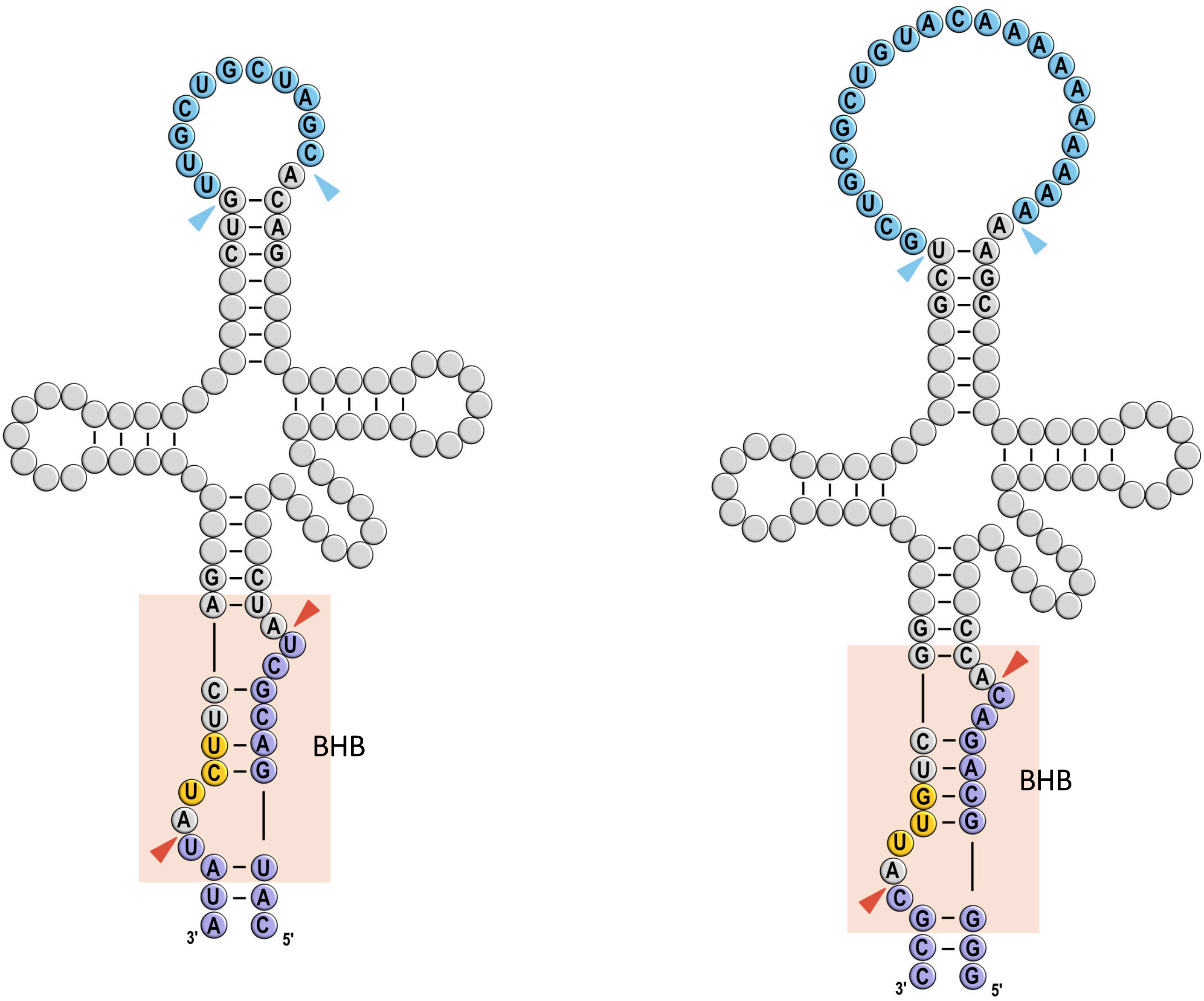

Appendix Figure S4. Secondary structures of *Chondrus crispus* pre-tRNAs. Secondary structures of pre-tRNA^{Tyr}, pre-tRNA^{Mete}, pre-tRNA^{Cys} and pre-tRNA^{Trp} with introns of archaeal-type are schematically shown. The CCA tri-nucleotides present at the 3' extremity are depicted with red circles, the anticodon with orange circles. Blue circles represent introns. Intron sequences and a few nucleotides of the mature tRNA sequences are indicated. BHB motifs of archaeal-type introns are under orange background. Arrowheads indicate cleavage sites. BHB: Bulge-Helix-Bulge.

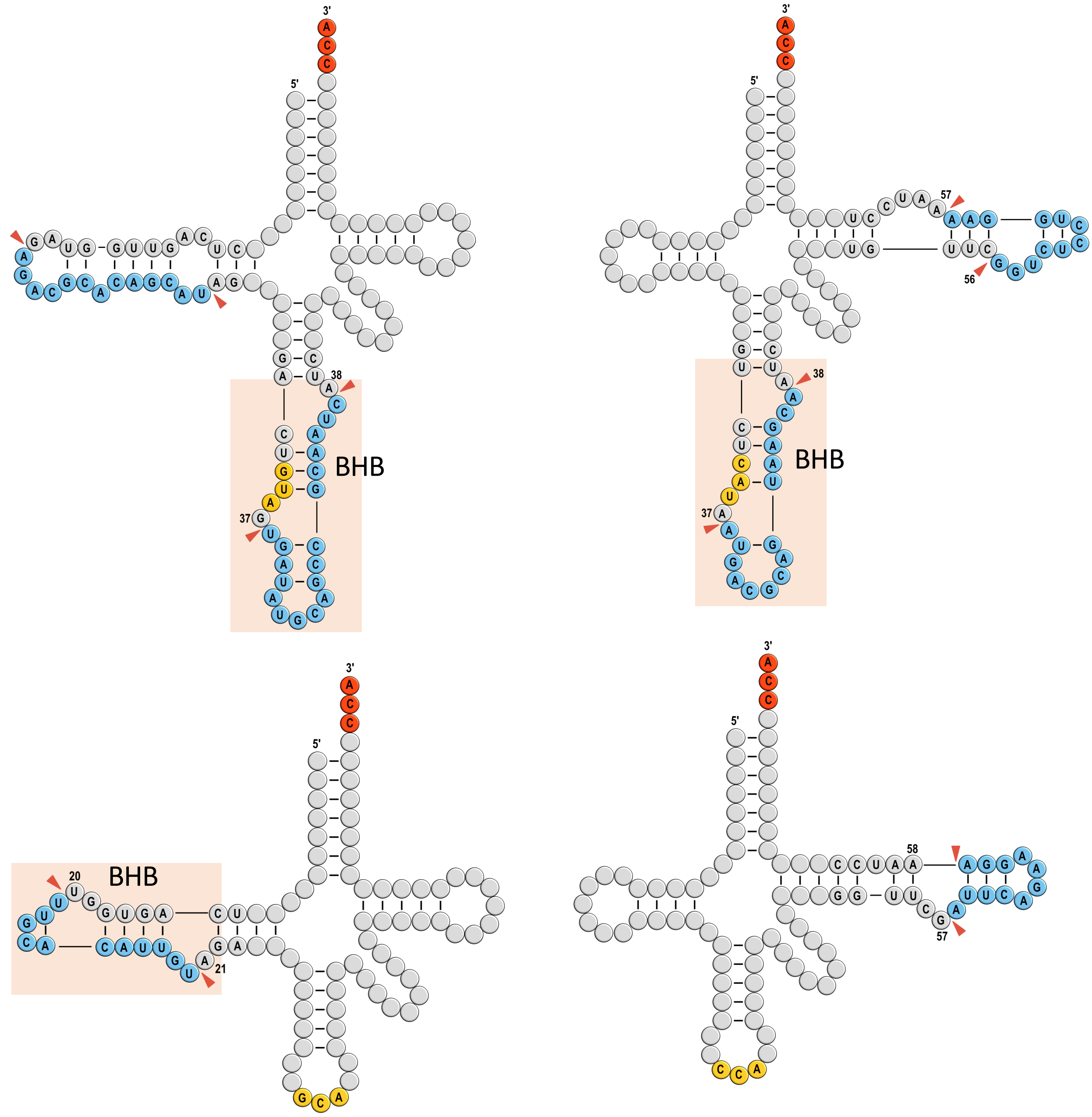

Appendix Figure S5. Secondary structures of *G. tetha* pre-tRNAs. Secondary structures of pre-tRNA^{Leu}, pre-tRNA^{Mete}, and pre-tRNA^{Asn} with introns are schematically shown. The CCA tri-nucleotides present at the 3' extremity are depicted with red circles, the anticodon with orange circles. Blue circles represent introns. Intron sequences and a few nucleotides of the mature tRNA sequences are indicated. Arrowheads indicate cleavage sites.

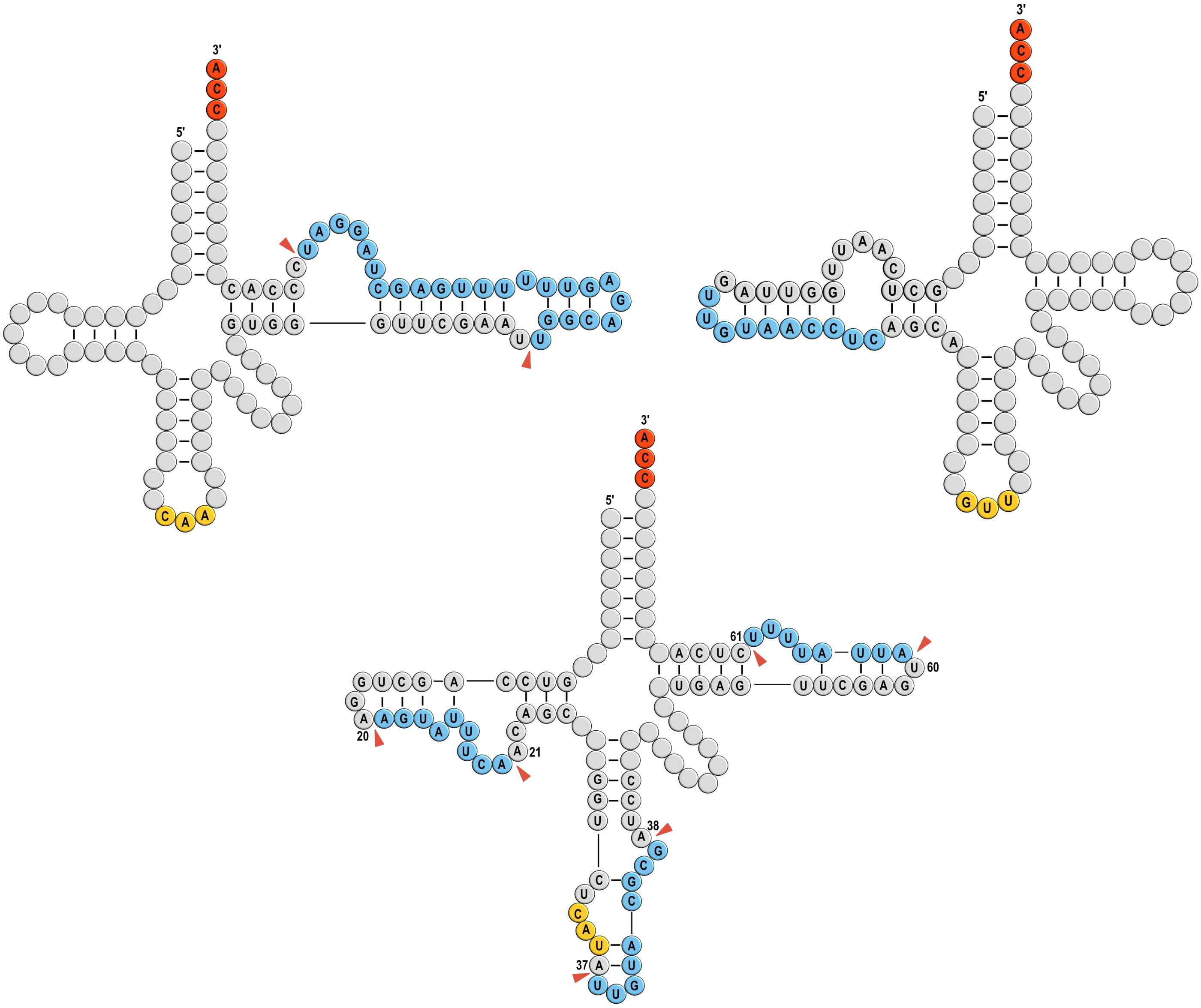

Appendix Figure S6. Organellar tRNA genes. **a**) Mitochondria-encoded tRNA genes. In orange, native tRNA genes, in green, plastid-like tRNA genes, and in blue tRNA genes of likely bacterial origin. Brown squares in Atr (*Amborella trichopoda*) represents the various origins of tRNA genes. * As a fern representative the mitochondrial tRNA gene population of *Ophioglossum californicum* (Oca) has been incorporated. **b**) Plastids-encoded tRNA genes. In green, native tRNA genes, in blue a tRNA gene of bacterial origin, in grey tRNA genes of unknown origin.
Names of organisms are abbreviated as in Table S1.

a

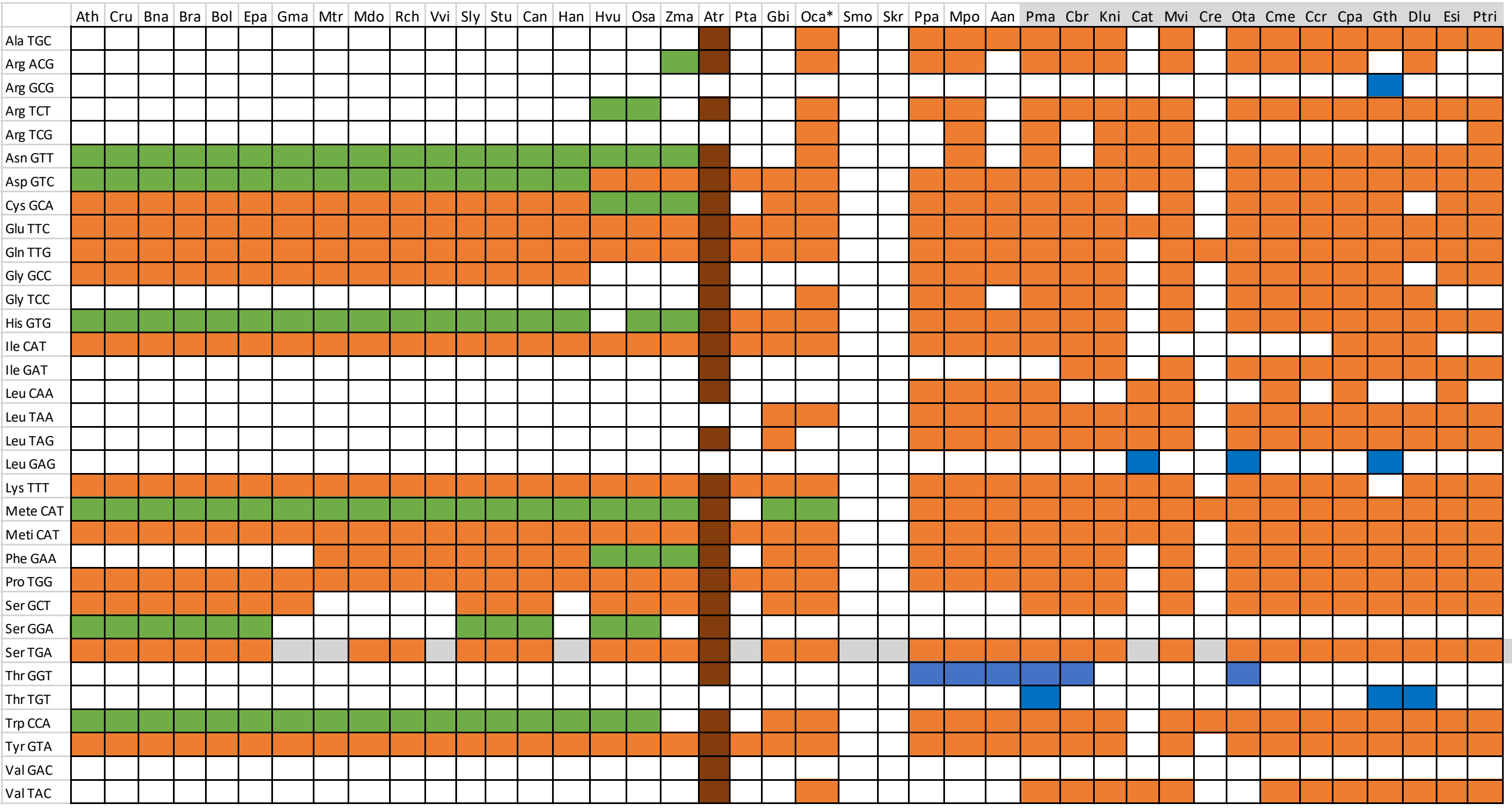

b

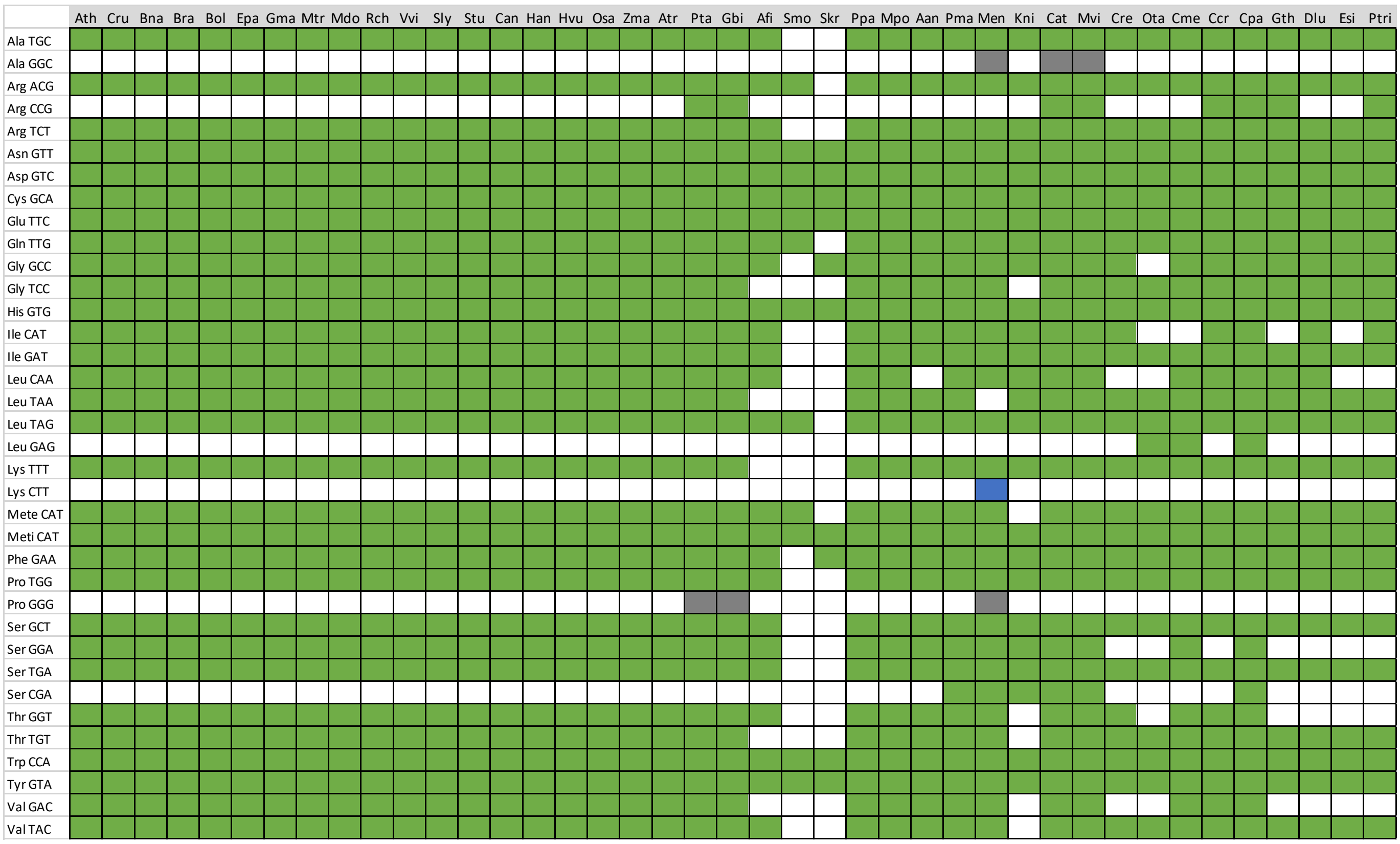

Appendix Figure S7. Mitochondrial tRNA genes of *Amborella trichopoda*. Number and identity of the 126 tRNA isoacceptor genes of different origins in the mitochondrial genome of *Amborella trichopoda*.

	Total	Algae	Moss	Plastid	Angiosperm	Amborella
Ala(TGC)	4	4				
Arg(ACG)	3	1	1	1		
Arg(TCT)	5	3	1	1		
Arg(TCG)	0					
Asn(GTT)	5	2		1	1	1
Asp(GTC)	8	3	1	3		1
Cys(GCA)	6	2	1		3	
Gln(TTG)	6	3	1		2	
Glu(TTC)	3	1		1	1	
Gly(UCC)	5	4	1			
Gly(GCC)	7	4	1	1	1	
His(GTG)	3	1				2
Ile(GAT)	2	2				
Ile(CAT)	4	4				
Leu(TAA)	0					
Leu(CAA)	6	5		1		
Leu(TAG)	6	4	1	1		
Lys(TTT)	10	2	3		3	2
Mete(CAT)	4			2	1	1
Meti(CAT)	8	1	2	1	3	1
Phe(GAA)	4			1	3	
Pro(TGG)	8	5	2			1
Ser(TGA)	3		2		1	
Ser(GCT)	3	1			2	
Ser(GGA)	1			1		
Thr(GGT)	1		1			
Thr(TGT)	0					
Trp(CCA)	2		1			1
Tyr(GTA)	3		1		2	
Val(TAC)	4	3	1			
Val(GAC)	2			2		
Total	126	55	21	17	23	10

Appendix Table S1: Photosynthetic species analyzed in this study.

Information regarding the number of nuclear, plastidial, and mitochondrial tRNA genes, as well as the number of nuclear and plastidial introns, was obtained from Cognat et al., 2022.

Clade	Species	Abbreviation	3 letters abbreviation	genome size (Mb)	Nuclear tRNA gene number	Plastidial tRNA gene number	Mt tRNA gene number	Nuclear tRNA isodecoders	% nuclear introns	% plastidial introns	Environment	Life cycle
Angiosperm	*Arabidopsis thaliana*	*A.thaliana*	Ath	135	585	37	22	174	13,8	21,6	Land	Annual
Angiosperm	*Arabidopsis lyrata*	*A. lyrata*	Aly	206,8	553	37	nd	229	9,3	nd	Land	Annual
Angiosperm	*Capsella rubella*	*C. rubella*	Cru	133,5	570	37	22	153	8,4	21,6	Land	Annual
Angiosperm	*Brassica napus*	*B. napus*	Bna	976,2	2214	36	17	517	7,4	22,2	Land	Annual
Angiosperm	*Brassica rapa*	*B.rapa*	Bra	353,2	1050	37	20	330	7,8	21,6	Land	Annual
Angiosperm	*Brassica oleracea*	*B.oleracea*	Bol	488,9	962	nd	35	457	6,4	nd	Land	Annual
Angiosperm	*Eutrema parvulum*	*E. parvulum*	Epa	137,5	586	38	17	179	5,3	21,1	Land	Annual
Angiosperm	*Eutrema salsigineum*	*E. salsigineum*	Esa	295,6	500	37	nd	194	9,6	21,6	Land	Annual
Angiosperm	*Populus trichocarpa*	*P. trichocarpa*	Ptr	434,3	505	37	nd	144	5,7	21,6	Land	Perenial
Angiosperm	*Phaseolus vulgaris*	*P. vulgaris*	Pvu	521,2	591	37	nd	251	4,9	21,6	Land	Annual
Angiosperm	*Glycine max*	*G. max*	Gma	978,9	700	37	20	180	5,4	21,6	Land	Annual
Angiosperm	*Medicago truncatula*	*M. truncatula*	Mtr	430	704	31	17	246	5,0	22,6	Land	Annual
Angiosperm	*Malus domestica*	*M.domestica*	Mdo	703,7	562	37	19	154	5,0	21,6	Land	Perenial
Angiosperm	*Fragaria iinumae*	*F. iinumae*	Fii	204,7	428	36	nd	143	4,7	19,4	Land	Perenial
Angiosperm	*Rosa chinensis*	*R. chinensis*	Rch	513,8	486	37	20	162	4,7	21,6	Land	Perenial
Angiosperm	*Vitis vinifera*	*V.vinifera*	Vvi	486,2	391	37	23	150	4,6	21,6	Land	Perenial
Angiosperm	*coffea canefora*	*C. canefora*	Cca	568,8	378	36	nd	147	4,2	19,4	Land	Perenial
Angiosperm	*Antirrhinum majus*	*A. majus*	Ama	500,8	579	37	nd	142	5,0	21,6	Land	Perenial
Angiosperm	*Primulina huijiensis*	*P. huijiensis*	Phu	470	293	36	nd	111	4,1	19,4	Land	Perenial
Angiosperm	*Solanum lycopersicon*	*S. lycopersicon*	Sly	828,3	723	37	26	253	5,9	21,6	Land	Annual
Angiosperm	*Solanum tuberosum*	*S. tuberosum*	Stu	706	738	37	29	209	6,0	21,6	Land	Annual
Angiosperm	*Nicotiana tabacum*	*N. tabacum*	Nta	3990	1230	37	22	410	6,2	21,6	Land	Annual
Angiosperm	*Capsicum annuum*	*C. annuum*	Can	2935,9	526	37	23	209	5,9	21,6	Land	Annual
Angiosperm	*Helianthus anuus*	*H. anuus*	Han	3027,8	924	36	18	236	5,1	22,2	Land	Annual
Angiosperm	*Hordeum vulgare*	*H. vulgare*	Hvu	4834,4	1009	38	28	258	5,6	21,1	Land	Annual
Angiosperm	*Brachypodium distachyon*	*B. distachyon*	Bdi	271,3	469	38	nd	124	5,5	21,1	Land	Annual
Angiosperm	*Oryza sativa*	*O. sativa*	Osa	374,4	543	36	30	171	6,1	16,7	Land	Annual
Angiosperm	*Zea mays*	*Z. mays*	Zma	2135,1	841	38	22	347	4,3	21,1	Land	Annual
Angiosperm	*Amborella trichopoda*	*A. trichopoda*	Atr	71	334	37	126	130	5,8	21,6	Land	Perenial
Gymnosperm	*Pinus taeda*	*P. taeda*	Pta	22105	1013	34	10	339	5,3	14,7	Land	Perenial
Gymnosperm	*Ginko biloba*	*G. biloba*	Gbi	9555,4	507	39	22	208	6,3	20,5	Land	Perenial
Fern	*Azolla filiculoides*	*A. filiculoides*	Afi	622,7	7848	32	nd	2943	5,7	21,9	Land	
Lycophyte	*Selaginella moellendorffii*	*S. moellendorffii*	Smo	213,2	920	14	0	454	4,7	0,0	Land	
Lycophyte	*Selaginella kraussiana*	*S. kraussiana*	Skr	114,5	456	10	0	277	5,7	0,0	Land	
Mosse	*Physcomitrium patens*	*P. patens*	Ppa	480,2	382	35	24	126	5,0	20,0	Land	
Liverwort	*Marchantia polymorpha*	*M. polymorpha*	Mpo	219	615	36	28	181	5,0	22,2	Land	
Hornwort	*Anthoceros angustus*	*A. angustus*	Aan	119,7	226	37	20	96	4,0	21,6	Land	
Streptophyte	*Penium margaritaceum*	*P. margaritaceum*	Pma	3662	1479	32	28	715	4,8	15,6	Aquatic	
Streptophyte	*Mesotaenium endlicherianum*	*M. endlicherianum*	Men	173,9	175	33	nd	74	18,9	3,0	Aquatic	
Streptophyte	*Spirogloea muscicola*	*S. muscicola*	Smu	170,8	152	nd	nd	64	8,0	nd	Aquatic	
Streptophyte	*Chara braunii*	*C. braunii*	Cbr	1751,5	373	nd	nd	137	7,2	nd	Aquatic	
Streptophyte	*Klebsormidium nitens*	*K. nitens*	Kni	104,2	112	35	nd	61	33,9	14,3	Aquatic	
Streptophyte	*Chlrorokybus atmophyticus*	*C. atmophyticus*	Cat	74,7	96	37	12	59	5,2	2,7	Aquatic	
Streptophyte	*Mesostigma viride*	*M. viride*	Mvi	441,9	249	36	26	61	18,1	0,0	Aquatic	
Chlorophyte	*Chlamydomonas reinhardtii*	*C. reinhardtii*	Cre	111,5	325	29	3	83	61,2	0,0	Aquatic	
Chlorophyte	*Ostrecococcus tauri*	*O. tauri*	Ota	13	45	27	28	43	48,9	0,0	Aquatic	
Rhodophyte	*Cyanidioschyzon merolae*	*C. merolae*	Cme	16,7	43	30	25	42	62,8	0,0	Aquatic	
Rhodophyte	*Chondrus crispus*	*C. crispus*	Ccr	105	103	30	23	53	35,2	3,3	Aquatic	
Glaucophyte	*Cyanophora paradoxa*	*C. paradoxa*	Cpa	100	139	36	30	47	5,0	2,8	Aquatic	
Cryptophyte	*Guillardia theta*	*G. theta*	Gth	87,8	123	29	26	58	14,5	0,0	Aquatic	
Haptophyte	*Diacronema lutheri*	*D. lutheri*	Dlu	43,6	56	27	24	43	0,0	0,0	Aquatic	
Heterokontophyte	*Ectocarpus siliculosus*	*E.siliculosus*	Esi	210,4	341	31	25	115	1,8	0,0	Aquatic	
Heterokontophyte	*Phaeodactylum tricornutum*	*P. tricornutum*	Ptri	19,7	52	30	23	42	19,2	0,0	Aquatic	
