# Supplementary material for: From water to land: evolution of tRNA gene repertoires in photosynthetic organisms
Source: EMBO J. 2026 Aug 22;45(18):6367–83. doi: 10.1038/s44318-026-00904-y (PMC13578239; doi:10.1038/s44318-026-00904-y)
Supplement: Supplementary file 2 [file 44318_2026_904_MOESM2_ESM.pdf]

THE EMBO JOURNAL

From water to land: evolution of tRNA gene repertoires in photosynthetic organisms

Guillaume Hummel, David Pflieger, Alexandre Berr, and Laurence Marechal-Drouard

Corresponding author(s): Laurence Marechal-Drouard (laurence.drouard@ibmp-cnrs.unistra.fr) , Alexandre Berr (alexandre.berr@ibmp-cnrs.unistra.fr)

Review Timeline:

Submission Date:	10th Nov 25
Editorial Decision:	11th Feb 26
Revision Received:	30th Apr 26
Editorial Decision:	2nd Jun 26
Revision Received:	5th Jun 26
Accepted:	11th Aug 26

Editor: Cornelius Schneider

Transaction Report:

(Note: With the exception of the correction of typographical or spelling errors that could be a source of ambiguity, letters and reports are not edited. Depending on transfer agreements, referee reports obtained elsewhere may or may not be included in this compilation. Referee reports are anonymous unless the Referee chooses to sign their reports.)

Dear Dr. Marechal-Drouard,

Thank you for submitting your manuscript for consideration by the EMBO Journal. It has now been seen by three referees whose comments are shown below.

As you can see from the reports, all three referees find the topic interesting, but they also note that the overall framing of the manuscript is at times inconsistent, as it blends meta-analysis of datasets with a review-style synthesis of the existing literature. In my assessment, the referees would be supportive of publication in The EMBO Journal if the manuscript were reframed as a review article.

Given that you have already indicated your willingness to undertake such revisions, I would like to invite you to submit a revised version of the manuscript addressing the comments of all three reviewers and adjusting the framing accordingly. Please note that The EMBO Journal allows only a single round of revision, and acceptance will therefore depend on the completeness and quality of your responses in this revised version.

When preparing your letter of response to the referees' comments, please bear in mind that this will form part of the Review Process File, and will therefore be available online to the community. For more details on our Transparent Editorial Process, please review our Editorial Policies: https://link.springer.com/partners/embo-press/editorial-policies

We generally allow three months as standard revision time. As a matter of policy, competing manuscripts published during this period will not negatively impact on our assessment of the conceptual advance presented by your study. However, we request that you contact the editor as soon as possible upon publication of any related work, to discuss how to proceed. Should you foresee a problem in meeting this three-month deadline, please let us know in advance and we may be able to grant an extension.

Thank you for the opportunity to consider your work for publication. I look forward to your revision.

Yours sincerely,

Cornelius Schneider, PhD
Editor
The EMBO Journal
c.schneider@embojournal.org

Instructions for preparing your revised manuscript:

Read our guidance for manuscript revisions and related editorial policies: https://link.springer.com/journal/44318/submission-guidelines#cms-Revised-submissions

Please make sure you upload a letter of response to the referees' comments together with the revised manuscript.

Please also check that the title and abstract of the manuscript are brief, yet explicit, even to non-specialists.

When assembling figures, please refer to our figure preparation guideline in order to ensure proper formatting and readability in print as well as on screen:
https://media.springernature.com/original/springer-cms/rest/v1/content/27825798/data/v1

At EMBO Press we ask authors to provide source data for the main manuscript figures. You will receive a separate email with instructions for providing source data with your revised manuscript, including how to upload and organize the files.

IMPORTANT: When you send the revision we will require
- a point-by-point response to the referees' comments, with a detailed description of the changes made (as a word file).
- a word file of the manuscript text.
- individual production quality figure files (one file per figure)
- a complete author checklist
- Expanded View files (replacing Supplementary Information)
- a Reagents and Tools Table as part of the Methods section

Please remember: Digital image enhancement is acceptable practice, as long as it accurately represents the original data and conforms to community standards. If a figure has been subjected to significant electronic manipulation, this must be noted in the figure legend or in the 'Materials and Methods' section. The editors reserve the right to request original versions of figures and the original images that were used to assemble the figure.

We realize that it is difficult to revise to a specific deadline. In the interest of protecting the conceptual advance provided by the work, we recommend a revision within 3 months (12th May 2026). Please discuss the revision progress ahead of this time with the editor if you require more time to complete the revisions. Use the link below to submit your revision:

https://emboj.msubmit.net/cgi-bin/main.plex

Referee #1:

Summary:
The authors present a comparative analysis of tRNA genes across the plant lineage, incorporating nuclear, plastid and mitochondrial genomes. They describe patterns of tRNA gain and loss, intron distribution, codon usage correlations and organellar tRNA evolution. Several observations - such as the stability of plastid tRNA sets versus the variability of mitochondrial sets, and the distribution of tRNASec - may be of interest to the community.
However, in its current form the manuscript lacks sufficient methodological transparency, analytical rigor and focus to support publication as an original research Article in The EMBO Journal. The work reads largely as a literature-driven review, with limited reproducible analysis and extensive speculative interpretation.

Major Points:
1. Lack of methodological description and reproducibility:
The manuscript does not contain a Methods section. Essential information - including dataset versions, annotation pipelines, software parameters and access to the described "shinytRNA" framework - is missing. Without these details, the analysis cannot be evaluated or reproduced.
2. Nature of the work resembles a review rather than original research:
The manuscript synthesizes previously published observations and provides narrative interpretation, but presents little clearly identifiable new analysis. Many claims refer to "our analyses" or "our dataset", but the underlying work is not documented. Several relevant recent studies on plant tRNA biology are not cited.
3. Unbalanced and unjustified taxon sampling:
The selection of 53 "representative" species is not explained and heavily overrepresents angiosperms, raising concerns about potential biases in comparative analyses.
4. Speculative interpretations not supported by data:
Several evolutionary and ecological claims (e.g. "preadaptation", "metabolic throughput", "intron loss facilitating land colonization') are presented without supporting evidence. Inferences based on tRNA intron positions to address deep evolutionary relationships are particularly problematic given the homoplasious nature of tRNA introns.
5. Insufficient statistical and phylogenetic rigor:
Cross-species analyses do not account for phylogenetic non-independence, invalidating correlation-based conclusions (e.g. Figure 5c). Sample sizes in key comparisons are small or unevenly distributed. P-values near 0.1 are presented as meaningful.
6. Organellar tRNA evolution treated descriptively:
The section summarizing organellar tRNA dynamics remains qualitative and literature-based. Quantitative phylogenetic analyses (e.g. ancestral state reconstruction, rate estimation) are needed to support the claims.
7. Issues of structure and focus:
The manuscript is unusually long and includes a broad introduction on RNA-world hypotheses that is only loosely connected to the presented results. The narrative frequently shifts between molecular, ecological and evolutionary levels, making the manuscript feel unfocused.

Minor Points:
1. Several figure legends are unclear or repetitive, and some figures are overly complex.
2. The manuscript occasionally confuses tRNA gene copy number with functional tRNA abundance.
3. The Results and Discussion sections are combined, making the logical flow difficult to follow.
4. Some concepts (e.g. tRNASec distribution, permuted tRNAs) are repeated across sections; consolidation would improve clarity.
5. The regression lines in Figure 5c are not justified by the reported R2 values; clarification is needed.

Overall Assessment:
The topic is interesting and relevant, but the manuscript requires substantial revision before it can be considered for publication in The EMBO Journal. The authors should provide a complete and transparent Methods section, clearly distinguish new results from literature synthesis, incorporate appropriate phylogenetic and statistical analyses and substantially focus the narrative. As

currently presented, the manuscript is not suitable for publication in this journal.

Referee #2:

This is a comprehensive treatise of tRNA genes in plants that underscores the main principles underlying their evolution. The three genetic systems of plants (nuclear/cytoplasmic, plastid, mitochondrial) stand in the foreground, as do the lack of organelle-encoded tRNA in mitochondria, requiring the import of tRNAs from the cytosol. There is no new data in the paper but the coverage of plant tRNA is the best and most up to date available. The Strasbourg team is the international leader in this field, so the paper comes from highest authority. That said, there is an appreciable case for making this interesting and important formally a review rather than an article.

Clearly, there is no translation without tRNA. But is it absolutely essential to couch the introduction in an "RNA world"? Arguably the paper is about tRNA in cellular evolution, not origin of life. For example Figure 1 goes from LUCA straight to algae with plastids acquired via secondary symbiosis, leaving out archaea and bacteria. One might want to highlight the archaeal and bacterial ancestry of cytosolic and organellar translation systems in Figure 1 as sketched in Figure 3. This could be worked up as a showcase figure.

Figure 1 has too many abbreviations, it is impossible for the average reader to decode the text-bits. Species names can be written radially. The reader needs more help on intron information. Where are they located in the genes (add a schematic to the figure)? What group-intron type? One tries to related the colors in B to those in A but there is no connection. In the "Imp" semicircle, there is room to expount the abbreviation "imported into mt" or similar; the green color intesity could be replaced by integers, a more specific information. B could be expanded by a structure.

While we are at figures, Figure 2c, give us some higher order taxa, please, I see Hsa and Cel so I assume there are animals in there as well as Ath plants. Figure 2b needs fiullaer explanation in the legend.

Figure 3a, the stars look like inventions, the interpretation is conservation, that could be symbolized by dotted lines.

Figure 4d, the red tRNAs are required under what kind of certain conditions, and do red and black both come grom the same gene? Symbiolism here is confusing for the generalist.

Figure 5b is extremely dense and seems to show the same pattern across all (undecipherable) species. Essentail? There is no panel 5c. Figure 5d: The message delivered by this observation is not clear. We see nonuniform distribution, but is it functionally significant? The different numbers of chromosomes make this a difficult interpretation. Figure 6a is more informative in this regard. There is enough room to write the species names and indicate higher taxa.

One could take issue with the old, wild-eyed claims for mitochondrion-to-mitochondrion gene transfer in plants, based on phylogenies that did not take nuclear pseudogenes or editing sites into account. Recall that land plant mtDNA almost does not evolve at all, such that editing sites change the phylogenies that were once seen as "evidence" for "LGT" [YE Lin, CS Wu, YW Wu, SM Chaw: Plants 14 (9), 1335 Phylogenomic inference suggests differential deep time phylogenetic signals from nuclear and organellar genomes in gymnosperms].

The text is by experts, there is not much room for critique. The text is perfectly suited for a review, which might suit this paper more readily than article. The figures are informative but contain too much symbolism, they could be altered to improve clarity.

Referee #3:

In the manuscript "From the RNA world to land plants: Evolutionary insights from tRNA genes", the authors present a comparative analysis of publicly available plant tRNA datasets (including 53 different species) to explore the hypothesis that tRNA gene evolution is a major determinant of translational capacity and a key driver of photosynthetic diversification. Overall, the article is well written, and the figures are well assembled. The manuscript contains a large amount of information and provides an excellent synthesis of what is currently known about tRNA biology in land plants, while introducing a comparative genomics approach that offers new insights into how tRNA evolution has shaped plant evolution.

Comments:

In general, the titles and subtitles could be revised to be more conclusion-driven rather than purely classificatory. This would

help readers more easily grasp the key messages and improve overall clarity. For example, instead of "From water to land, expansion and diversification of tRNA gene repertoires in photosynthetic eukaryotes," the title could be "tRNA gene number scales loosely with organismal complexity and genome dynamism, but no single factor fully explains the observed variation." Similarly, "Family- and lineage-specific clustering patterns" could be rephrased as "tRNA clustering patterns are family- and lineage-specific."

Figure 1: I find this figure highly informative and visually appealing. My only suggestion would be to add a color-coded graphical legend in the center of the cladogram. I would also recommend choosing color combinations that avoid reds and greens to improve accessibility for color-deficient readers.

Please cite Table S1 near the sentence "Variation is equally striking at the genomic scale," as some of the information discussed there is summarized in that table.
In Table S1, do the authors refer to the percentage of tRNAs with nuclear or plastidial introns in the last two columns? If so, does the "-" symbol indicate the complete absence of introns, or simply a lack of evidence?

Page 3: The authors hypothesize that tRNA number might influence growth speed, or vice versa, although a direct causal link remains to be demonstrated. However, I do not see any duckweed species (family Lemnaceae) included in their analysis. I suggest adding some of these species to strengthen this argument, or at least mentioning them as a future perspective.

Page 4: There are a few typographical issues here, specifically brackets that open but do not close. Please check and correct these.

Supplemental Table 1: Please include information indicating which species were classified as annual or perennial, and as aquatic or terrestrial plants, since these classifications were used in some of the analyses presented in Figure 2.

I believe Figure 2b could be removed. It is not self-explanatory and is difficult to interpret, and I am not convinced it adds value beyond what is already stated in the main text: "To ensure optimal protein synthesis, tRNAs decoding frequently used codons must not be limiting, whereas excessive tRNAs for rare codons could compromise translational fidelity and generate misfolded or deleterious proteins." Removing Figure 2b would also allow Figure 2c to be expanded, making it possible to increase the font size of the organism names.

It would be useful for readers to include a schematic representation of permutated tRNAs in Supplementary Figure 4.

Figure 4b: The tRNA gene is not drawn to scale relative to the upstream and downstream sequences. Please indicate this either in the figure legend or directly in the schematic.

Figure 4c: Please specify in the figure legend that the species names corresponding to groups A-E are provided in Supplementary Figure 8.

Regarding cis-regulatory elements of tRNAs, I wonder whether there are differences between solo and clustered tRNAs. These sequences may differ in their significance between these two contexts. Have the authors performed any analyses addressing this question?

The authors mention the resource (https://nebula.ibmp.unistra.fr/shinytRNA/
); however, I did not find a table or clear access point for it, and there is no citation provided. Please correct this.

Figure S12: I suggest generating the same figure but excluding tRNA clusters, analogous to what was done for Arabidopsis in the right panel of Figure 5a.

Response to reviewers (EMBO Journal MS# 2024-12221)

Referee #1 (Report for Author)

Summary:
The authors present a comparative analysis of tRNA genes across the plant lineage, incorporating nuclear, plastid and mitochondrial genomes. They describe patterns of tRNA gain and loss, intron distribution, codon usage correlations and organellar tRNA evolution. Several observations - such as the stability of plastid tRNA sets versus the variability of mitochondrial sets, and the distribution of tRNASec - may be of interest to the community.
However, in its current form the manuscript lacks sufficient methodological transparency, analytical rigor and focus to support publication as an original research Article in The EMBO Journal. The work reads largely as a literature-driven review, with limited reproducible analysis and extensive speculative interpretation.

Major Points
1. Lack of methodological description and reproducibility:
The manuscript does not contain a Methods section. Essential information - including dataset versions, annotation pipelines, software parameters and access to the described "shinytRNA" framework - is missing. Without these details, the analysis cannot be evaluated or reproduced.
We fully agree with the reviewer that the previous version inappropriately combined original meta-analysis with a literature-driven review, without providing a dedicated Material and Methods section. In response, we substantially restructured the work by clearly separating the original comparative meta-analysis from the review manuscript. The meta-analysis of datasets has been removed from the main manuscript and is now presented as an arXiv preprint (arXiv:2511.01943), which includes detailed methodological descriptions, analytical pipelines, and the ShinytRNA resource. The revised manuscript submitted to The EMBO Journal is now explicitly framed as a review article focused on the synthesis and critical interpretation of current knowledge on tRNA gene evolution.

2. Nature of the work resembles a review rather than original research:
The manuscript synthesizes previously published observations and provides narrative interpretation, but presents little clearly identifiable new analysis. Many claims refer to "our analyses" or "our dataset", but the underlying work is not documented. Several relevant recent studies on plant tRNA biology are not cited.
As noted above, we agree with the reviewer that this work is best framed as a review. We have updated the bibliography throughout the manuscript and incorporated several recent studies relevant to plant tRNA biology and genome organization where appropriate (e.g. Zhang et al, 2025; Mohanta et al, 2020, Szymanski et al, 2023).

3. Unbalanced and unjustified taxon sampling: The selection of 53 "representative" species is not explained and heavily overrepresents angiosperms, raising concerns about potential biases in comparative analyses.
As noted above, the meta-analysis on the 53 species is now part of the arXiv preprint. In that work, the rationale for taxon selection is explicitly described and reflects the availability of high-quality chromosome-level genome assemblies and reliable tRNA annotations across major photosynthetic lineages. The stronger representation of angiosperms mainly reflects the current imbalance in genomic resources available across photosynthetic eukaryotes rather than a deliberate sampling bias.

4. Speculative interpretations not supported by data: Several evolutionary and ecological claims (e.g. "preadaptation", "metabolic throughput", "intron loss facilitating land colonization') are presented without supporting evidence. Inferences based on tRNA intron positions to address deep evolutionary relationships are particularly problematic given the homoplasious nature of tRNA introns.

We agree with the reviewer that several evolutionary interpretations in the previous version were presented too strongly despite remaining speculative. Accordingly, the review has been revised to clearly distinguish supported observations from hypotheses, particularly regarding preadaptation to terrestrialization, links between tRNA gene number and growth-related traits, and the evolutionary significance of intron loss. We also reduced overinterpretation concerning deep phylogenetic inference based on tRNA intron positions and now present these patterns more cautiously as suggestive rather than demonstrative.

5. Insufficient statistical and phylogenetic rigor: Cross-species analyses do not account for phylogenetic non-independence, invalidating correlation-based conclusions (e.g. Figure 5c). Sample sizes in key comparisons are small or unevenly distributed. P-values near 0.1 are presented as meaningful.
As the manuscript has now been reframed as a review article, Figure 5 and the associated meta-analyses have been removed and are now presented separately in the arXiv preprint.

6. Organellar tRNA evolution treated descriptively: The section summarizing organellar tRNA dynamics remains qualitative and literature-based. Quantitative phylogenetic analyses (e.g. ancestral state reconstruction, rate estimation) are needed to support the claims.
We agree with the reviewer that the section on mitochondrial and plastid tRNA gene evolution is primarily based on existing literature rather than new quantitative phylogenetic analyses. This is consistent with the revised framing of the manuscript as a review article rather than an original research study. We agree that approaches such as ancestral state reconstruction or evolutionary rate estimation could offer valuable insights into organellar tRNA gene evolution. However, such analyses are beyond the scope of the present review and would be more appropriately addressed in dedicated comparative studies.

7. Issues of structure and focus: The manuscript is unusually long and includes a broad introduction on RNA-world hypotheses that is only loosely connected to the presented results. The narrative frequently shifts between molecular, ecological and evolutionary levels, making the manuscript feel unfocused.
We thank the reviewer for this important comment and agree that the previous version was too broad in scope and insufficiently focused. The introduction has been substantially shortened and the broad introduction on RNA world hypotheses has been removed to better align the manuscript with its central focus on tRNA gene evolution in photosynthetic eukaryotes We also reorganized several sections to improve the overall narrative flow and reduce unnecessary shifts between molecular, ecological, and evolutionary levels of interpretation.

Minor Points:
1. Several figure legends are unclear or repetitive, and some figures are overly complex.
Figures related to the comparative meta-analysis (Figures 2b, 2c, 4b, 4c, 5, and 6a) have been removed, and the corresponding legends have been revised accordingly. We also simplified the remaining figures and legends to improve clarity, readability, and reduce redundancy.

2. The manuscript occasionally confuses tRNA gene copy number with functional tRNA abundance.
We agree with the reviewer that the distinction between tRNA gene copy number and tRNA abundance was not always sufficiently clear. We have carefully revised the manuscript throughout to eliminate this ambiguity.

3. The Results and Discussion sections are combined, making the logical flow difficult to follow.
We thank the reviewer for this comment. As the manuscript has now been restructured as a review article, the distinction between Results and Discussion sections is no longer applicable.

4. Some concepts (e.g. tRNASec distribution, permuted tRNAs) are repeated across sections; consolidation would improve clarity.
We thank the reviewer for this constructive comment. In the revised review, we substantially reduced the overall length of the manuscript and reorganized several sections to consolidate overlapping content

particularly regarding tRNASec distribution and atypical tRNA architectures. This helped minimize repetition. and improve overall clarity and readability.

5. The regression lines in Figure 5c are not justified by the reported R2 values; clarification is needed.
As the manuscript has now been reframed as a review article, Figure 5 and the associated metadata-analyses have been removed from the revised version of the review.

Referee #2 (Report for Author)

This is a comprehensive treatise of tRNA genes in plants that underscores the main principles underlying their evolution. The three genetic systems of plants (nuclear/cytoplasmic, plastid, mitochondrial) stand in the foreground, as do the lack of organelle-encoded tRNA in mitochondria, requiring the import of tRNAs from the cytosol. There is no new data in the paper but the coverage of plant tRNA is the best and most up to date available. The Strasbourg team is the international leader in this field, so the paper comes from highest authority. That said, there is an appreciable case for making this interesting and important formally a review rather than an article.
We thank the reviewer for this positive assessment and fully agree that the manuscript is more appropriately framed as a review rather than an original research article. Accordingly, the revised version has been substantially restructured as a review, with the comparative meta-analysis separated into an independent arXiv preprint and the main manuscript now focused on the synthesis and interpretation of current knowledge on tRNA gene evolution in photosynthetic organisms.

Clearly, there is no translation without tRNA. But is it absolutely essential to couch the introduction in an "RNA world"? Arguably the paper is about tRNA in cellular evolution, not origin of life. For example Figure 1 goes from LUCA straight to algae with plastids acquired via secondary symbiosis, leaving out archaea and bacteria. One might want to highlight the archaeal and bacterial ancestry of cytosolic and organellar translation systems in Figure 1 as sketched in Figure 3. This could be worked up as a showcase figure.
We thank the reviewer for this insightful comment and agree that the primary focus of the manuscript is tRNA gene evolution in photosynthetic organisms rather than the origin of life. In response, we have revised the introduction to remove the broader “RNA world” framing and now focus more on plant evolution and tRNAs diversification.
We also appreciate the reviewer’s constructive suggestions regarding Figure 1. The figure has been revised to better illustrate the archaeal and bacterial ancestry of cytosolic and organellar translation systems through a simplified schematic. This now provides a clearer evolutionary framework and improves consistency with the concepts developed later in the manuscript.

Figure 1 has too many abbreviations, it is impossible for the average reader to decode the text-bits. Species names can be written radially. The reader needs more help on intron information. Where are they located in the genes (add a schematic to the figure)? What group-intron type? One tries to related the colors in B to those in A but there is no connection. In the "Imp" semicircle, there is room to expount the abbreviation "imported into mt" or similar; the green color intensity could be replaced by integers, a more specific information. B could be expanded by a structure.
We thank the reviewer for highlighting the complexity of Figure 1 and for the constructive suggestions to improve its readability. In response, we have revised the figure to enhance clarity for a broader audience. Abbreviations have been minimized by replacing the three-letter species codes with more explicit names, and the different semi-circles are now more clearly defined to guide interpretation.
To further improve visual accessibility, we have adopted the colorblind-friendly *Dark2* palette. In addition, color gradients have been replaced or complemented by more explicit quantitative indicators, including color ladders within the figure that specify the proportion of tRNAs containing nuclear or plastidial introns, as well

as the percentage of tRNAs predicted to be imported into mitochondria. These legends are now directly integrated into the figure for ease of reference.
We agree that additional information on intron location and group types would be valuable. However, incorporating this level of detail into Figure 1 would considerably increase its complexity and reduce overall readability. Instead, these aspects are described in detail in the main text (pages 5-8) and illustrated in Figures 2b, 2c, and Supplementary Figures S3–S5.
Finally, to address the reviewer's suggestion regarding structural representation, Figure 2b has been designed to provide complementary insight, including consistent color coding across three representations of a mature tRNA: a 2D structure, a 3D model, and a ribbon diagram.

While we are at figures, Figure 2c, give us some higher order taxa, please, I see Hsa and Cel so I assume there are animals in there as well as Ath plants. Figure 2b needs fiullaer explanation in the legend.
Figures 2b and 2c have been removed from the revised version. As a result, the specific concerns regarding taxonomic labeling in Figure 2c and the level of detail in the legend of Figure 2b are no longer directly applicable to the revised manuscript. For completeness, we note that in a parallel version available on the arXiv open-access repository (*aRxiv:2511.01943)*, Figure 2c has been updated and non-plant abbreviations are explicitly defined to improve clarity.

Figure 3a, the stars look like inventions, the interpretation is conservation, that could be symbolized by dotted lines.
We thank the reviewer for this insightful comment and agree that the use of stars was potentially misleading, as it could be interpreted as indicating novel features rather than conservation. In our original figure, the stars were intended to highlight the presence of non-canonical introns; however, we recognize that this representation was not sufficiently clear.
In the revised version, we have removed the stars. Using dotted lines was considered, but the result was ambiguous. Therefore, we choose to indicate the presence of non-canonical introns using a schematic tRNA representation enclosed within a circle, which we believe provides a clearer and more intuitive visualization of these features.

Figure 4d, the red tRNAs are required under what kind of certain conditions, and do red and black both come grom the same gene? Symbiolism here is confusing for the generalist.
We thank the reviewer for pointing out the lack of clarity in the symbolism used in Figure 4d (now Figure 3a in the revised version). We agree that the distinction between red and black tRNAs, including the conditions under which the red tRNAs are required and their relationship to the same gene, was not sufficiently explained and could be confusing for a general audience. To address this issue and improve overall clarity, we simplified the figure by removing the red tRNAs and depicting only black tRNAs. We believe this revision provides a clearer and more accessible representation of the intended message.

Figure 5b is extremely dense and seems to show the same pattern across all (undecipherable) species. Essentail? There is no panel 5c. Figure 5d: The message delivered by this observation is not clear. We see nonuniform distribution, but is it functionally significant? The different numbers of chromosomes make this a difficult interpretation. Figure 6a is more informative in this regard. There is enough room to write the species names and indicate higher taxa.
We agree with the reviewer that these figures were too dense and that the biological message was not sufficiently clear in the original version. As part of the restructuring of the manuscript into a review article, Figure 5b and Figure 6a have been removed and are now presented in the arXiv version (*aRxiv:2511.01943)*, where they are discussed in a more appropriate analytical context.

One could take issue with the old, wild-eyed claims for mitochondrion-to-mitochondrion gene transfer in plants, based on phylogenies that did not take nuclear pseudogenes or editing sites into account. Recall that land plant mtDNA almost does not evolve at all, such that editing sites change the phylogenies that were once

seen as "evidence" for "LGT" [YE Lin, CS Wu, YW Wu, SM Chaw: Plants 14 (9), 1335 Phylogenomic inference suggests differential deep time phylogenetic signals from nuclear and organellar genomes in gymnosperms].
We agree that caution is required when interpreting mitochondrial phylogenetic analyses in plants, particularly given potential confounding factors such as RNA editing sites, nuclear pseudogene contributions, and the extremely slow rate of mitochondrial genome evolution in land plants. These factors can indeed influence phylogenetic signal and have, in some cases, led to overinterpretation of ancient horizontal transfer events. We have now revised the manuscript to explicitly acknowledge these limitations and to moderate the interpretation of putative mitochondrion-to-mitochondrion transfer events. We also incorporated the suggested reference (page 8).

The text is by experts, there is not much room for critique. The text is perfectly suited for a review, which might suit this paper more readily than article. The figures are informative but contain too much symbolism, they could be altered to improve clarity.
We thank the reviewer for this positive assessment. As suggested, the revised version is now presented as a review article, and the remaining figures have been substantially simplified and clarified as described above.

Referee #3 (Report for Author)

In the manuscript "From the RNA world to land plants: Evolutionary insights from tRNA genes", the authors present a comparative analysis of publicly available plant tRNA datasets (including 53 different species) to explore the hypothesis that tRNA gene evolution is a major determinant of translational capacity and a key driver of photosynthetic diversification. Overall, the article is well written, and the figures are well assembled. The manuscript contains a large amount of information and provides an excellent synthesis of what is currently known about tRNA biology in land plants, while introducing a comparative genomics approach that offers new insights into how tRNA evolution has shaped plant evolution.

Comments:

In general, the titles and subtitles could be revised to be more conclusion-driven rather than purely classificatory. This would help readers more easily grasp the key messages and improve overall clarity. For example, instead of "From water to land, expansion and diversification of tRNA gene repertoires in photosynthetic eukaryotes," the title could be "tRNA gene number scales loosely with organismal complexity and genome dynamism, but no single factor fully explains the observed variation." Similarly, "Family- and lineage-specific clustering patterns" could be rephrased as "tRNA clustering patterns are family- and lineage-specific."
We thank the reviewer for this helpful suggestion. As the manuscript has now been reframed as a review article, we retained most section titles to preserve a clear and structured thematic organization. However, several titles and subtitles were revised for improved clarity and to better reflect the main conceptual messages of each section.

Figure 1: I find this figure highly informative and visually appealing. My only suggestion would be to add a color-coded graphical legend in the center of the cladogram. I would also recommend choosing color combinations that avoid reds and greens to improve accessibility for color-deficient readers.
We thank the reviewer for the valuable comment. As indicated above to reviewer 2, the quality of Figure 1 has been improved: a color-coded graphical legend was added in the center of the cladogram and a colorblind-friendly *Dark2* palette chosen to improve visual accessibility to color-deficient readers.

Please cite Table S1 near the sentence "Variation is equally striking at the genomic scale," as some of the information discussed there is summarized in that table.

In Table S1, do the authors refer to the percentage of tRNAs with nuclear or plastidial introns in the last two columns? If so, does the "-" symbol indicate the complete absence of introns, or simply a lack of evidence?
We thank the reviewer for this helpful suggestion. In response, we have now cited Table S1 at the relevant point in the Introduction, specifically in the sentence "At the genomic scale, this diversity ..." where it provides supporting information.
We also thank the reviewer for pointing out the need for clarification regarding Table S1. The last two columns of the previous version indeed refer to the percentage of tRNA genes containing nuclear or plastidial introns. To improve clarity, this information is now explicitly stated in the column headers of the corresponding columns. In addition, we apologize for the lack of clarity regarding the symbol "–", which originally indicated missing information. This has now been replaced by "nd" ("not determined") to avoid ambiguity.

Page 3: The authors hypothesize that tRNA number might influence growth speed, or vice versa, although a direct causal link remains to be demonstrated. However, I do not see any duckweed species (family Lemnaceae) included in their analysis. I suggest adding some of these species to strengthen this argument, or at least mentioning them as a future perspective.
We thank the reviewer for this insightful suggestion and agree that duckweeds (Lemnaceae), as some of the smallest and fastest-growing angiosperms, represent a particularly relevant system for exploring the proposed relationship between tRNA gene content and growth-related traits. While a direct analysis of duckweed species is not included in the present study, we have now added a statement highlighting their relevance as a promising future direction for testing this hypothesis. This point is now mentioned in the revised manuscript (page 4), together with the corresponding reference (Ernst et al., 2025).

Page 4: There are a few typographical issues here, specifically brackets that open but do not close. Please check and correct these.
Typographical issues have been corrected.

Supplemental Table 1: Please include information indicating which species were classified as annual or perennial, and as aquatic or terrestrial plants, since these classifications were used in some of the analyses presented in Figure 2.
Information indicating which species were classified as annual or perennial, and as aquatic or terrestrial plants has now been added to Table S1 for clarity and completeness.

I believe Figure 2b could be removed. It is not self-explanatory and is difficult to interpret, and I am not convinced it adds value beyond what is already stated in the main text: "To ensure optimal protein synthesis, tRNAs decoding frequently used codons must not be limiting, whereas excessive tRNAs for rare codons could compromise translational fidelity and generate misfolded or deleterious proteins." Removing Figure 2b would also allow Figure 2c to be expanded, making it possible to increase the font size of the organism names.
We thank the reviewer for this helpful suggestion. We agree that Figure 2b was not essential for supporting the main message of the manuscript and may not have been sufficiently self-explanatory. It has therefore been removed in the revised version to improve clarity and streamline the presentation.
We also appreciate the suggestion regarding Figure 2c. While it is not included in the revised manuscript now presented as a review, it has been retained in a parallel, openly accessible version available on arXiv (arXiv:2511.01943), where it is presented as part of a broader meta-analysis.

It would be useful for readers to include a schematic representation of permutated tRNAs in Supplementary Figure 4.
A schematic representation of two permuted introns of O. tauri pre-tRNAs is now presented in Supplemental Figure S3.

Figure 4b: The tRNA gene is not drawn to scale relative to the upstream and downstream sequences. Please indicate this either in the figure legend or directly in the schematic.

We thank the reviewer for pointing out this issue. To avoid possible confusion, we revised the figure (now Figure 3a) by introducing a clear visual break (white interruption) within the tRNA gene representation to explicitly indicate that the scale is not continuous.

Figure 4c: Please specify in the figure legend that the species names corresponding to groups A-E are provided in Supplementary Figure 8.
In the revised manuscript, Figure 4c has been removed. As part of the comparative meta-analysis, Figure 4c is now presented in the arXiv version where the figure legend has been updated to explicitly indicate that the species names corresponding to groups A–E are provided in a supplemental figure.

Regarding cis-regulatory elements of tRNAs, I wonder whether there are differences between solo and clustered tRNAs. These sequences may differ in their significance between these two contexts. Have the authors performed any analyses addressing this question?
We thank the reviewer for this very interesting question. In the comparative meta-analysis presented separatly in the arXiv version (arXiv:2511.01943), we examined whether cis-regulatory elements differ between clustered and dispersed tRNA genes. We observed that clustered tDNAs tend to show reduced cis-regulatory divergence compared to dispersed loci, suggesting stronger local conservation of their regulatory environment. However, we did not detect a clear correlation between the overall enrichment of cis-elements and whether a tRNA gene occurs as a singleton or within a cluster.
As this analysis belongs to the comparative meta-analysis rather than the review itself, the detailed results are presented in the arXiv version. However, we now briefly mention this observation in the revised review to clarify that clustering does not appear to systematically alter the overall enrichment of canonical Pol III regulatory motifs.

The authors mention the resource ((https://nebula.ibmp.unistra.fr/shinytRNA/) however, I did not find a table or clear access point for it, and there is no citation provided. Please correct this.
We thank the reviewer for identifying this issue. The ShinytRNA resource is now explicitly included and described in the arXiv version. The resource is properly referenced, and the access link (https://nebula.ibmp.unistra.fr/shinytRNA/) has been verified and updated to ensure clear availability.

Figure S12: I suggest generating the same figure but excluding tRNA clusters, analogous to what was done for Arabidopsis in the right panel of Figure 5a.
Figure S12 has been removed as it was part of the Meta-analysis.

Dear Dr. Marechal-Drouard,

Thank you for submitting your manuscript for consideration by the EMBO Journal. It has now been seen by two of the original referees whose comments are enclosed. As you will see, the referees express interest in your manuscript and are broadly in favor of publication, pending satisfactory additional revision requested by referee #1 which we think are reasonable and productive.

When preparing your letter of response to the referees' comments, please bear in mind that this will form part of the Review Process File, and will therefore be available online to the community. For more details on our Transparent Editorial Process, please review our Editorial Policies page: https://link.springer.com/partners/embo-press/editorial-policies

We generally allow three months as standard revision time. As a matter of policy, competing manuscripts published during this period will not negatively impact on our assessment of the conceptual advance presented by your study. However, we request that you contact the editor as soon as possible upon publication of any related work, to discuss how to proceed.

Thank you for the opportunity to consider your work for publication. I look forward to your revision.

Yours sincerely,

Cornelius Schneider, PhD
Editor
The EMBO Journal
c.schneider@embojournal.org

Instructions for preparing your revised manuscript:

Read our guidance for manuscript revisions and related editorial policies: https://link.springer.com/journal/44318/submission-guidelines#cms-Revised-submissions

Please also check that the title and abstract of the manuscript are brief, yet explicit, even to non-specialists.

When assembling figures, please refer to our figure preparation guideline in order to ensure proper formatting and readability in print as well as on screen:
https://media.springernature.com/original/springer-cms/rest/v1/content/27825798/data/v1

IMPORTANT: When you send the revision we will require
- a point-by-point response to the referees' comments, with a detailed description of the changes made (as a word file).
- a word file of the manuscript text.
- individual production quality figure files (one file per figure)
- a complete author checklist
- Expanded View files (replacing Supplementary Information)
- a Reagents and Tools Table as part of the Methods section

Please remember: Digital image enhancement is acceptable practice, as long as it accurately represents the original data and conforms to community standards. If a figure has been subjected to significant electronic manipulation, this must be noted in the figure legend or in the 'Methods' section. The editors reserve the right to request original versions of figures and the original images that were used to assemble the figure.

We realize that it is difficult to revise to a specific deadline. In the interest of protecting the conceptual advance provided by the work, we recommend a revision within 3 months (31st Aug 2026). Please discuss the revision progress ahead of this time with the editor if you require more time to complete the revisions. Use the link below to submit your revision:

https://emboj.msubmit.net/cgi-bin/main.plex

Referee #1:

The authors have made substantial and largely appropriate revisions. The manuscript is now clearly framed as a review, not original research. The tone is more cautious, the speculative claims have been significantly reduced and the structure is much improved. However, some critical issues remain that I would like to point out.

1. The current version of the manuscript still refers to "our analyses" or "our dataset" (e.g., page 3: "We first explored..."; page 9: "Analysis of upstream... revealed..."). I am missing a clear sentence in the main text stating that quantitative analyses are detailed in the preprint. Currently, a reader might still think that the review contains primary data.
2. The manuscript makes general statements about "photosynthetic eukaryotes" or "land plants" based on examples that are predominantly angiosperms. For example: (1) page 3: "Terrestrial plants display substantially larger repertoires" - supported by box plots in Figure 1c, but those data presumably come from the 53-species set. Without seeing the taxonomic breakdown in the preprint, the reviewer cannot verify whether non-angiosperm land plants are adequately represented; (2) page 4: fast-growing crops vs. moderate growers - all represented by angiosperms. In my opinion the text should explicitly acknowledge the sampling bias.
3. I have found some speculative statements: e.g., on page 6: "P. margaritaceum... may represent a preadaptation toward rapid and robust tRNA maturation under fluctuating conditions" and: "intron reduction predates the emergence of land plants, although the evolutionary forces... remain poorly understood and difficult to disentangle". Such statements in my opinion should be clearly labeled as hypothetical.
4. The section about the organellar tRNA evolution remains weak for a review. It is largely a catalog of which lineages lost which tRNA genes, with little synthesis or critical evaluation. One would expect: (1) a clearer comparative table of mitochondrial vs. plastid tRNA retention across major lineages (not just text); (2) discussion of why mitochondria lose tRNAs faster than plastids - not just "instability"; (3) a more critical take on evidence for import (e.g., how do we know imported tRNAs are functional?). The section on Amborella (page 8) is interesting but looks like an isolated example.

Additional issues:
1. The review repeatedly cites Cognat et al. 2022 for tRNA numbers, intron presence, etc. and independent validation from other studies is rarely mentioned. A strong review would note potential annotation biases (e.g., tRNAscan-SE parameters, misannotation of pseudogenes).
2. The manuscript covers glaucophytes, red algae, green algae, land plants, and secondary endosymbionts. But euglenids and chlorarachniophytes are mentioned only briefly. The title and abstract should reflect the primary focus on Archaeplastida and derived lineages. Currently, it implies a broader coverage than delivered.
3. Given that tRNA modifications are central to function and show lineage-specific patterns, their absence in a review on tRNA evolution is notable. The authors mention pseudouridylation in tRNA Tyr (page 6) but this subject is not systematically and sufficiently addressed.
4. In addition, I would welcome the addition of a "Limitations and perspectives" section at the end of the review, where all the limitations and biases of this study could be explicitly listed.

Referee #3:

I think the authors have addressed my comments and concerns. I also agree that this paper is more suitable for a review article style.

Response to reviewers (EMBOJ-2025-123003R)

Referee #1 (Report for Author)

The authors have made substantial and largely appropriate revisions. The manuscript is now clearly framed as a review, not original research. The tone is more cautious, the speculative claims have been significantly reduced and the structure is much improved. However, some critical issues remain that I would like to point out.

We sincerely thank Referee #1 for the positive assessment of our revisions. We are pleased that the reframing as a review, the more cautious tone, and the improved structure have been well received. We have carefully addressed the remaining concerns raised below. A copy of the manuscript with changes in red has been included.

1. The current version of the manuscript still refers to "our analyses" or "our dataset" (e.g., page 3: "We first explored..."; page 9: "Analysis of upstream... revealed..."). I am missing a clear sentence in the main text stating that quantitative analyses are detailed in the preprint. Currently, a reader might still think that the review contains primary data.

We thank the referee for this precise observation. We have revised all such instances to use clearly attributive phrasing (e.g. page 3 and 10).

2. The manuscript makes general statements about "photosynthetic eukaryotes" or "land plants" based on examples that are predominantly angiosperms. For example: (1) page 3: "Terrestrial plants display substantially larger repertoires" - supported by box plots in Figure 1c, but those data presumably come from the 53-species set. Without seeing the taxonomic breakdown in the preprint, the reviewer cannot verify whether non-angiosperm land plants are adequately represented; (2) page 4: fast-growing crops vs. moderate growers - all represented by angiosperms. In my opinion the text should explicitly acknowledge the sampling bias.

We thank the referee for raising this point. We fully acknowledge that the dataset underlying our comparisons is enriched in angiosperms relative to other land plant groups, which is an inherent consequence of current genome availability rather than a deliberate choice. We have added an explicit caveat at the first mention of Figure 1c (page 3) and have softened the language in the fast-growing crops paragraph (page 4) to acknowledge that all examples cited are angiosperms. This limitation is also explicitly listed in the new Limitations and perspectives section added at the end of the review.

3. I have found some speculative statements: e.g., on page 6: "P. margaritaceum... may represent a preadaptation toward rapid and robust tRNA maturation under fluctuating conditions" and: "intron reduction predates the emergence of land plants, although the evolutionary forces... remain poorly understood and difficult to disentangle". Such statements in my opinion should be clearly labeled as hypothetical.

We thank the referee for these precise observations. We have added explicit hypothetical markers to the two flagged sentences. We note that the overall tone of the manuscript already relies extensively on cautious language which we believe is appropriate for a review article integrating comparative genomic evidence.

4. The section about the organellar tRNA evolution remains weak for a review. It is largely a catalog of which lineages lost which tRNA genes, with little synthesis or critical evaluation. One would expect: (1) a clearer comparative table of mitochondrial vs. plastid tRNA retention across major lineages (not just text); (2) discussion of why mitochondria lose tRNAs faster than plastids - not just "instability"; (3) a more critical take

on evidence for import (e.g., how do we know imported tRNAs are functional?). The section on Amborella (page 8) is interesting but looks like an isolated example.

(1) We agree that a visual synthesis would strengthen this section. Following the reviewer's suggestion, we will add a comparative figure displaying plastid tRNA gene retention across major lineages, analogous to the existing Supplementary Figure S6 for mitochondria, and place both panels side by side (now Figure S6a and S6b).
(2) We have added a short paragraph explicitly addressing this contrast (page 8), discussing the role of structural genome rearrangements in accelerating mitochondrial gene loss (Sloan et al., 2012) and the tighter transcriptional constraints acting on plastid genomes (Zoschke & Bock, 2018) as key contributing factors to this asymmetry.
(3) We have added a short paragraph clarifying the nature of evidence typically used to support tRNA import, and noting the limits of inference based solely on genomic absence. In the case of Amborella, we have also better integrated this example into the broader discussion of mitochondrial tRNA mosaicism, using it explicitly to illustrate the difficulty of distinguishing functional genes from pseudogenes in highly chimeric mitochondrial genomes.

Additional issues:
1. The review repeatedly cites Cognat et al. 2022 for tRNA numbers, intron presence, etc. and independent validation from other studies is rarely mentioned. A strong review would note potential annotation biases (e.g., tRNAscan-SE parameters, misannotation of pseudogenes).

We appreciate this comment. The current literature on plant tRNA genes remains relatively limited, and consequently a small number of studies, including Cognat et al. (2022), provide much of the available comparative data on tRNA gene numbers, intron content, and related features. Nevertheless, we agree that annotation-derived estimates should be interpreted with caution, as they may be influenced by methodological factors such as the choice of tRNA prediction software, parameter settings, and the potential mis-annotation of pseudogenes or highly divergent tRNA sequences. To address this important point, we have added a discussion of these potential annotation biases and their implications in the “Limitations and Perspectives” section of the review.

2. The manuscript covers glaucophytes, red algae, green algae, land plants, and secondary endosymbionts. But euglenids and chlorarachniophytes are mentioned only briefly. The title and abstract should reflect the primary focus on Archaeplastida and derived lineages. Currently, it implies a broader coverage than delivered.

We thank the reviewer for this comment. We agree that the review does not provide an equally detailed treatment of all photosynthetic eukaryotic lineages. Our primary focus is on Archaeplastida, for which comparative genomic data on tRNA gene repertoires are most abundant. However, we also include representative examples from lineages derived from secondary endosymbiosis whenever relevant data are available. For example, chlorarachniophytes are represented by *Bigelowiella natans* (Figure 2c).
A more comprehensive analysis of all photosynthetic lineages is currently limited by the availability of published genomic analyses and curated tRNA annotations. This is particularly true for euglenids, where genomic resources remain scarce and tRNA annotations are still incomplete. Nevertheless, we agree that this lineage deserves mention because it presents an intriguing contrast: its plastid genome retains a complete set of tRNA genes (Hallick et al., 1993), whereas no mitochondrially encoded tRNA genes have been identified (Dobáková et al., 2015). This last information is now presented page 8.
While we acknowledge that Archaeplastida constitute the core of the review, we believe the current title remains appropriate because the review also discusses representative secondary plastid-bearing lineages.

3. Given that tRNA modifications are central to function and show lineage-specific patterns, their absence in

a review on tRNA evolution is notable. The authors mention pseudouridylation in tRNA Tyr (page 6) but this subject is not systematically and sufficiently addressed.

We fully understand the referee's expectation. However, this review focuses deliberately on tRNA gene evolution at the genomic level (copy number, gene structure, introns, cis-regulatory elements, and chromosomal organisation). tRNA modifications constitute a vast and rapidly expanding field that would require a dedicated review to address systematically. The mention of pseudouridylation at U35 of tRNATyr is included because it is directly mechanistically linked to the tRNATyr intron, which is central to our intron section. We have made this editorial scope decision explicit in the Limitations and perspectives section, with pointers to dedicated recent reviews on tRNA modifications.

4. In addition, I would welcome the addition of a "Limitations and perspectives" section at the end of the review, where all the limitations and biases of this study could be explicitly listed.
A “limitations and perspectives” section has been added at the end of the review.

Dear Dr. Marechal-Drouard,

I am pleased to inform you that your manuscript has been accepted for publication in the EMBO Journal.

Your manuscript will be processed for publication by EMBO Press. It will be copy edited and you will receive page proofs prior to publication. Please note that you will be contacted by Springer Nature Author Services to complete licensing and payment information.

You may qualify for financial assistance for your publication charges - either via a Springer Nature fully open access agreement or an EMBO initiative. Check your eligibility: https://link.springer.com/journal/44318/how-to-publish-with-us

Should you be planning a Press Release on your article, please notify the production team via this form in order to coordinate publication and release dates: https://forms.cloud.microsoft/e/eH4A8JVP6g

If you have any questions, please do not hesitate to contact the Editorial Office. Thank you for your contribution to The EMBO Journal.

Yours sincerely,

Cornelius Schneider, PhD
Editor
The EMBO Journal
c.schneider@embojournal.org

*****
Please note that it is The EMBO Journal policy for the transcript of the editorial process (containing referee reports and your response letters) to be published as an online supplement to each paper. If you should prefer removal of any referee-only figures included in the point-by-point response(s), e.g. because they may still be used for future publication or because they have been reproduced from published work by others, please do let us know immediately via response email.
More information is available here: https://link.springer.com/partners/embo-press/editorial-policies#Peer%20review

** Click here to be directed to your login page: https://emboj.msubmit.net
